# Heterostructured Bismuth Telluride Selenide Nanosheets for Enhanced Thermoelectric Performance

**DOI:** 10.1002/smsc.202000021

**Published:** 2020-10-25

**Authors:** Christoph Bauer, Igor Veremchuk, Christof Kunze, Albrecht Benad, Volodymyr M. Dzhagan, Danny Haubold, Darius Pohl, Gabi Schierning, Kornelius Nielsch, Vladimir Lesnyak, Alexander Eychmüller

**Affiliations:** ^1^ Physical Chemistry TU Dresden Zellescher Weg 19 01069 Dresden Germany; ^2^ Max Planck Institute of Chemical Physics of Solids Nöthnitzer Str. 40 01187 Dresden Germany; ^3^ Semiconductor Physics Chemnitz University of Technology Reichenhainer Str. 70 09126 Chemnitz Germany; ^4^ Institute of Semiconductor Physics National Academy of Sciences of Ukraine Nauky av. 45 03028 Kyiv Ukraine; ^5^ Dresden Center for Nanoanalysis TU Dresden Helmholtzstraße 18 01069 Dresden Germany; ^6^ Leibniz Institute for Solid State and Materials Research Dresden Helmholtzstraße 20 01069 Dresden Germany; ^7^ Institute of Applied Physics TU Dresden Nöthnitzer Str. 61 01187 Dresden Germany; ^8^ Institute of Materials Science TU Dresden Helmholtzstr. 7 01069 Dresden Germany

**Keywords:** bismuth chalcogenides, colloidal synthesis, core/shell heterostructures, nanosheets, thermoelectrics

## Abstract

The n‐type semiconductor system Bi_2_Te_3_—Bi_2_Se_3_ is known as a low‐temperature thermoelectric material with a potentially high efficiency. Herein, a facile approach is reported to synthesize core/shell heterostructured Bi_2_Te_2_Se/Bi_2_Te_3_ nanosheets (NSs) with lateral dimensions of 1–3 μm and thickness of about 50 nm. Bi_2_Te_3_ and Bi_2_Se_3_, as well as heterostructured Bi_2_Te_2_Se/Bi_2_Te_3_ NSs are obtained via colloidal synthesis. Heterostructured NSs show an inhomogeneous distribution of the chalcogen atoms forming selenium and tellurium‐rich layers across the NS thickness, resulting in a core/shell structure. Detailed morphological studies reveal that these structures contain nanosized pores. These features contribute to the overall thermoelectric properties of the material, inducing strong phonon scattering at grain boundaries in compacted solids. NSs are processed into nanostructured bulks through spark plasma sintering of dry powders to form a thermoelectric material with high power factor. Electrical characterization of our materials reveals a strong anisotropic behavior in consolidated pellets. It is further demonstrated that by simple thermal annealing, core/shell structure can be controllably transformed into alloyed one. Using this approach pellets with Bi_2_Te_2.55_Se_0.45_ composition are obtained, which exhibit low thermal conductivity and high power factor for in‐plane direction with *zT* of 1.34 at 400 K.

## Graphical abstract

Bi_2_Te_3_, Bi_2_Se_3_, and core/shell Bi_2_Te_2_Se/Bi_2_Te_3_ nanosheets with lateral dimensions of 1–3 μm and thickness of about 50 nm are obtained via colloidal synthesis. Heterostructured nanosheets show an inhomogeneous distribution of the chalcogen atoms and nanosized pores. They are processed into nanostructured bulks with Bi_2_Te_2.55_Se_0.45_ composition, exhibiting low thermal conductivity and high power factor with *zT* of 1.34 at 400 K.

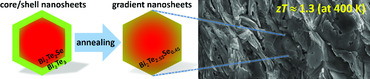

## 1 Introduction

The field of thermoelectrics received great attention with the progressing development of new materials and the rising global demand for cost‐effective, pollution‐free technologies of energy conversion. Thermoelectric materials enable the conversion of a heat flux into electricity (Seebeck effect) and vice versa (Peltier effect).^[^ ^1^ ^]^ Their perspective fields of application are, for example, in cooling devices^[^ ^2^ ^]^ or in waste heat recovery,^[^ ^3^ ^]^ making them attractive from economic and ecological points of view. Their advantages compared with other energy conversion or cooling devices include the absence of moving parts and zero emission of CO_2_ and/or hazardous substances.^[^ ^4^ ^]^ One of the requirements for a wide application of these materials is their further optimization to maximize the output. The efficiency of a thermoelectric material is given by the dimensionless figure of merit *zT* ^[^ ^1^ ^]^

(1) $$zT=\frac{S^{2}\sigma }{\kappa }T$$

where *S* is the Seebeck coefficient, *σ* is the electrical conductivity, *T* is the absolute temperature, and *κ* is the thermal conductivity. According to this equation, reducing the thermal conductivity, while keeping high electrical conductivity and the Seebeck coefficient, is the way for improving the *zT* value. The thermal conductivity *κ* is represented by the sum of the electronic thermal conductivity *κ*_el_ and the lattice thermal conductivity *κ*_l_. In addition, at higher temperatures, for narrow bandgap semiconductors or semimetals, the bipolar contribution of the thermal conductivity plays a significant role in the phonon and electron transport.^[^ ^5^ ^]^ The reduction of the phonon and bipolar components can have remarkable impact on the final *zT* value. The task of optimizing these main parameters is extremely challenging because they are interdependent. So, careful adjustment of different material properties has to be taken into account.

Different approaches have been used so far to increase the power factor *S* ^2^ *σ* of thermoelectric materials by means of, e.g., energy filtering,^[^ ^6^ ^]^ effective mass tuning,^[^ ^7^ ^]^ crystal structure engineering through its disordering by the formation of solid solutions,^[^ ^8^ ^]^ or band convergence.^[^ ^9^ ^]^ On the contrary, building of hierarchical architectures^[^ ^10^ ^]^ and nanoengineering^[^ ^11^, ^12^, ^13^ ^]^ have been used extensively to lower the lattice thermal conductivity. Generally, the lattice part of the thermal conductivity is defined by the sum of the contributions from the different phonon frequencies.^[^ ^14^ ^]^ The transport of phonons is affected by three factors: Umklapp processes, scattering from point defects, and grain boundaries.^[^ ^8^, ^10^ ^]^ In addition, other lattice imperfections such as interphases, intergrowths, dislocations, and impurities can also reduce phonon transport. The elimination of the bipolar effect can be realized by several strategies: increasing the majority carrier density,^[^ ^5^ ^]^ enlarging the bandgap,^[^ ^5^, ^15^ ^]^ building up an interfacial potential energy barrier through nanostructured boundaries,^[^ ^16^, ^17^ ^]^ or inducing selective scattering of minority carriers and thus limiting their conductivity.^[^ ^18^ ^]^

Nanostructuring, as one of the methods of reducing *κ*_l_, has proven to be very effective for various systems.^[^ ^19^ ^]^ Thus, with the introduction of nanosized grain boundaries in a thermoelectric material primarily low and intermediate energy phonons are scattered. Theoretical calculations account approximately 80% of the lattice thermal conductivity to the intermediate frequency phonons, showing the importance of their efficient scattering to achieve overall low *κ*.^[^ ^20^, ^21^ ^]^ An additional advantage of using polycrystalline materials is that they are more resilient than brittle single crystals. As thermoelectrics, usually semiconductors containing heavy elements and possessing a narrow band gap are exploited, as they already exhibit a low lattice thermal conductivity and a high carrier mobility, such as Bi_2_Te_3_ and PbTe.^[^ ^22^ ^]^ Among a wide variety of thermoelectrics, developed and studied during the last decades, the binary bismuth chalcogenides Bi_2_Te_3_ and Bi_2_Se_3_, as well as their solid solutions, are the most efficient converters of temperature gradients into electricity in the low‐temperature regime. In particular, Bi_2_Te_3_ is one of the most efficient low‐temperature thermoelectric materials and it is used in modern thermoelectric cooling units.^[^ ^23^ ^]^ Recently, the interest in these well‐known thermoelectrics was revitalized due to the discovery of a new form of quantum matter, namely, the 3D topological insulators.^[^ ^24^, ^25^, ^26^, ^27^ ^]^ This new quantum matter should be a key for the good thermoelectric properties, although the direct influence has not yet been confirmed experimentally and comprehensively investigated.

To improve *zT* of n‐type bismuth chalcogenide‐based materials, doping, alloying, and nanostructuring have been used. Recent results have shown that high *zT* values can be achieved with n‐type Bi_2_Te_3_‐based nanocrystals, grown in wet‐chemical synthesis. For example, solvothermally synthesized Bi_2_Te_3−*x*_Se_*x*_ alloy nanosheets (NSs) exhibit a *zT* of 1.23 at 480 K.^[^ ^11^ ^]^ Recently, Liu et al. demonstrated that *zT* of 1.31 at 438 K can be achieved for Bi_2_Te_2.7_Se_0.3_ NSs with the incorporation of Te nanorods.^[^ ^19^ ^]^ These results, in both cases showing very distinct nanocrystals of Bi_2_Te_3—*x*_Se_*x*_, emphasize the importance of opening more synthetic routes to nanostructured n‐type Bi_2_Te_3_‐based materials to understand and improve the material properties. An example for this is the elaborated synthesis of multishell (Bi_2_Se_3_)_*m*_/(Bi_2_Te_3_)_*n*_ NSs by Min et al., in which hydroxylamine was used to induce controllable nucleation as well as seed growth.^[^ ^28^ ^]^ This report showed an increase in *zT* to moderate 0.71 in contrast to nanocrystalline Bi_2_Te_3_. Thereafter, additional efforts to produce core/shell NSs were made by Li et al. synthesizing Bi_2_Te_2.7_Se_0.3_/(Bi_2_Te_3_)_*m*_.^[^ ^29^ ^]^ The overall performance was enhanced to *zT* of 1.17 by keeping extremely low thermal conductivity after sintering. Unfortunately, both syntheses are not a one‐pot method, and by this they are less suitable for upscaling and further industrial application.

In this study, we developed a facile synthesis of Bi_2_Te_2_Se/Bi_2_Te_3_ (Bi_2_Te_3—*x*_Se_*x*_) core/shell NSs with single‐crystalline orientation. These materials were obtained using a colloidal heating‐up approach, yielding large NSs with lateral dimensions of up to 3 μm and thicknesses of 6–60 nm depending on the composition. Among them the heterostructured NSs exhibited peculiar morphological features, such as nanosized holes. The additional substructuring was considered as an effective way to further decrease their thermal conductivity. Subsequently, the as‐synthesized NSs in the form of dry powders were heat‐treated to remove organic surface capping ligands and consolidated into nanostructured bulk pellets by spark plasma sintering (SPS). Owing to this nanoengineering, *zT* value of 0.85 (cross‐plane) for n‐type heterostructured Bi_2_Te_3—*x*_Se_*x*_ NSs with a relatively high selenium content (about 9 at%) was achieved at 440 K. We observed strong anisotropy from 2D core/shell Bi_2_Te_2_Se/Bi_2_Te_3_ heterostructures with reduced electrical conductivity measured parallel to the pressing direction. Low thermal conductivities were preserved even after a long annealing, and controllable alloying over the course of 24 h into nearly fully alloyed NSs was observed by heat treatment at 350 °C. Thermoelectric characterization in in‐plane direction showed high *zT* value over 1.3 at 400 K as a consequence of low thermal conductivity achieved through efficient nanostructuring.

## 2 Results and Discussion

Within this work we show the colloidal synthesis of heterostructured Bi_2_Te_3—*x*_Se_*x*_ NSs, followed by their processing *via* thermal treatment to remove residual organics and compaction through SPS into dense pellets with subsequent characterization of their thermal conductivity via laser flash analysis (LFA), followed by cutting into bars, contacting them, and performing in‐plane electrical measurements. Cross‐plane electrical characterization was performed on 3 mm‐thick pellets (for details of the experiments, see the Supporting Information).

### 2.1 Morphological and Structural Characterization of Bismuth Chalcogenide NSs

**Figure 1:**
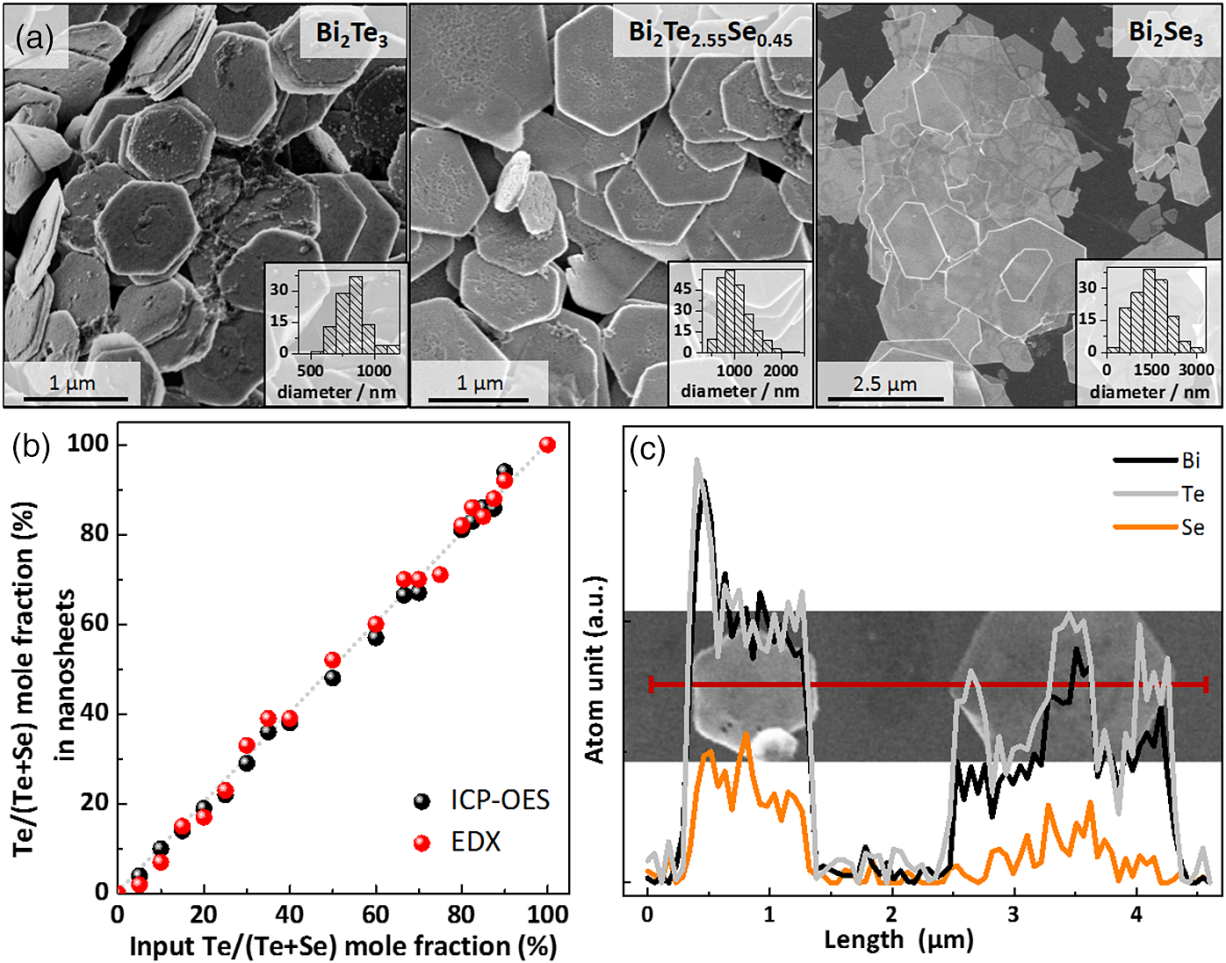
a) SEM images of binary and ternary NSs. b) Elemental composition of the synthesized alloyed bismuth chalcogenide NSs versus the ratio between Te and Se precursors in the reaction mixture, and c) the line spectrum resolved EDS of two single alloyed NSs synthesized using Te:Se ratio of 9:1.

**Figure** **1** shows scanning electron microscopy (SEM) images and the corresponding size distributions of hexagonal Bi_2_Te_3_, Bi_2_Se_3_, and heterostructured Bi_2_Te_3—*x*_Se_*x*_ NSs, synthesized via a facile, easily upscalable and reproducible method. An advantage of this synthetic procedure is that the reaction between the precursors proceeds under ambient atmosphere and thus does neither require a degassing step under vacuum nor the usage of inert gases. Furthermore, upscaling can be performed without any changes in the precursor ratios. Commonly, we scaled the original recipe^[^ ^30^ ^]^ 15‐fold to produce ≈0.75 g of the product. As shown in the SEM images in Figure 1a, all three types of NSs exhibit a well‐defined hexagonal 2D shape with lateral dimensions of ≈700–900 nm for Bi_2_Te_3_, 1000–2000 nm for Bi_2_Se_3_, and 700–1000 nm for Bi_2_Te_3—*x*_Se_*x*_. The average thickness of the sheets was 20—30 nm for Bi_2_Te_3_ and 6—12 nm for Bi_2_Se_3_. The elemental content of the binary and ternary phases was determined via an inductively coupled plasma optical emission spectroscopy (ICP‐OES) and an energy dispersive X‐ray spectroscopy (EDS) and found to be stoichiometric. The samples are named after their total composition, e.g., NSs obtained using a Te:Se ratio of 85:15 will be termed Bi_2_Te_2.55_Se_0.45_. The ternary Bi_2_Te_3—*x*_Se_*x*_ NSs were characterized by means of SEM, EDS, and ICP‐OES to determine how the particle size changes with the composition and what is the relation between the feed precursor ratio and the elemental content in the resulting NSs, as shown in Figure 1b. As follows from the data shown in Figure 1b and Table SI1, Supporting Information, the actual chemical composition of the NSs only slightly deviates from the initial ratio between the precursors. Therefore, they react quantitatively providing a simple means to control the final composition of the materials by changing the feed ratio. Furthermore, by transmission electron microscopy (TEM)–EDS analysis we confirmed a nonuniform distribution of all three elements observable from the top‐view of a single NS and from the side.

As shown in Figure 1a, the ternary alloy NSs possess a size distribution positioned between the two binary compounds, indicating an influence of the precursors and their content on the growth kinetics. When the elemental ratio of Te:Se precursors was above 2:1, the NSs grew hexagonal in shape (more SEM images of samples are shown in Figure SI4, Supporting Information). Below this ratio, we observed twinned and elongated hexagon‐like NSs. At higher magnifications, on the surface of heterostructured NSs irregularities can be seen (Figure SI5, Supporting Information). These NSs appear to have small pores, a structure which can be beneficial for the overall thermoelectric performance of the material.

**Figure 2:**
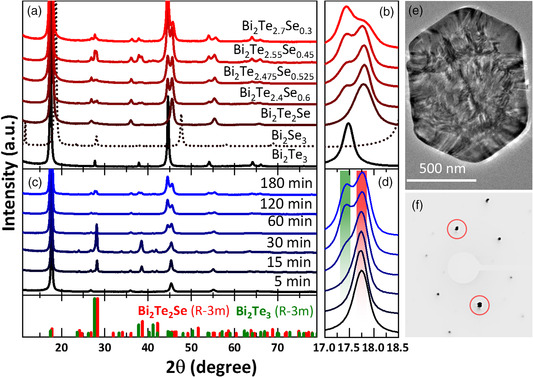
XRD patterns of Bi_2_Te_3_, Bi_2_Se_3_, Bi_2_Te_2_Se alloy, and heterostructures. a) Overview measurement for all colloidally synthesized samples, and b) with magnified splitting of the reflexes between 17.0° and 18.5° 2*θ*. c) XRD patterns of Bi_2_Te_2.55_Se_0.45_ NS samples taken after different reaction times with d) magnified splitting of the reflexes from the (006) plane between 17.0 and 18.5 2*θ*°. e) TEM image of a ternary Bi_2_Te_2.55_Se_0.45_ NS with visible moiré fringes and f) diffraction pattern with highlighted double spots, indicating the existence of two crystal phases in one NS.

X‐ray diffraction (XRD) of the purified reaction products obtained with Te:Se ratios higher than 2:1 revealed the existence of two crystal phases of rhombohedral Bi_2_Te_2_Se and Bi_2_Te_3_ (**Figure** **2**a,b). XRD characterization of the Bi_2_Te_2.55_ Se_0.45_ NS samples taken at different reaction times was performed to study the formation of the two crystal phases (Figure 2c,d). Preparation of the specimens via drop‐casting NSs dispersions in *iso‐*propanol (*i*PrOH) resulted in a preferred NS orientation along the (001) crystal plane that influences the relative signal intensities of the sample which can be seen, e.g., in the dominant (006) reflex centered at 17–18.5° 2*θ* (Figure 2a,b). In the diffractograms, the Se‐containing Bi_2_Te_2_Se alloy species were found already after 5 min of the reaction at 190 °C. The intensity of the reflexes attributed to this crystal phase increases with time (from 5 to 30 min). Thereafter, the reflexes of the Bi_2_Te_3_ crystal structure appear as a shoulder. Consequently, the development of the pure Bi_2_Te_3_ phase results in peak splitting (Figure 2b). In addition, we monitored the growth of the sheets via SEM/EDS during the reaction. By reaching 190 °C porous plates preformed. In these particles, an elemental ratio of roughly Bi_2_Te_2_Se was measured (results of elemental analysis *via* EDS are presented in Figure SI6, Supporting Information), in agreement with the XRD data. In the time range between 5 min and 2 h after reaching 190 °C, tellurium rods were observed in SEM images. As these rods are not present in the final product, we conclude that they are further reduced to form Bi_2_Te_3_. After 2 h reaction time, large NSs with holey structure developed, and after 3 h, full‐grown NSs were obtained.

**Figure 3:**
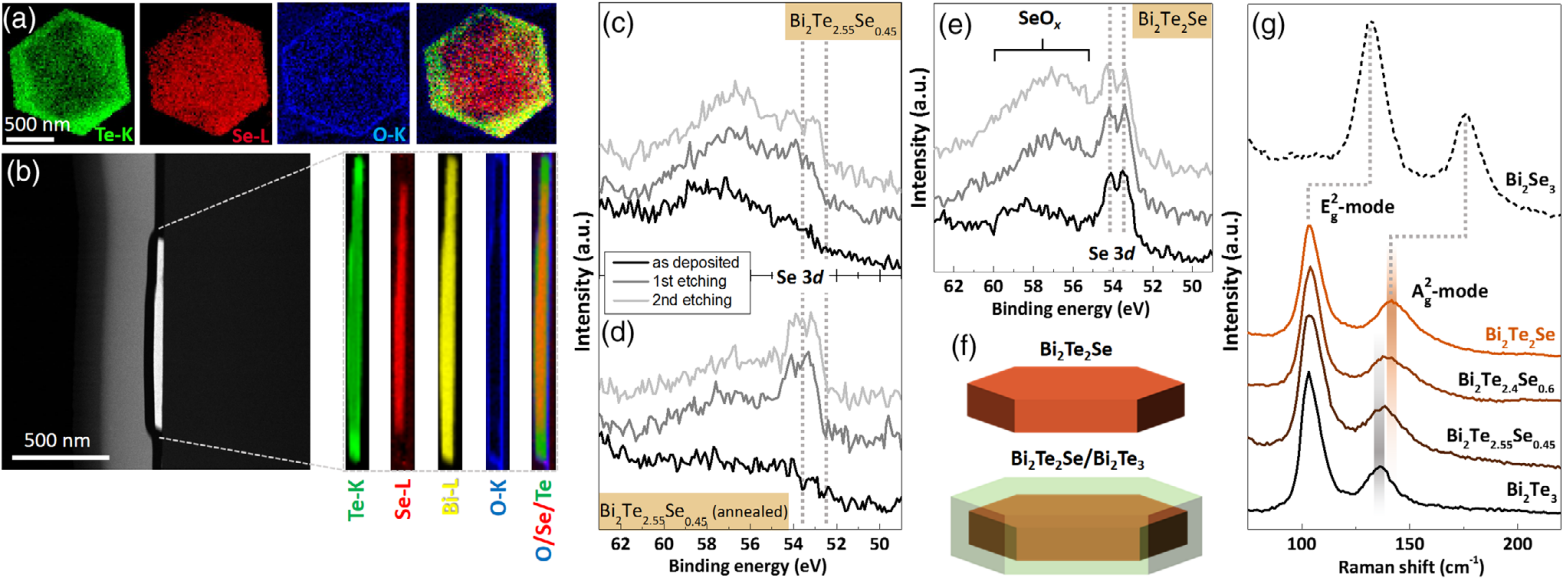
Elemental mapping of a single Bi_2_Te_2.55_Se_0.45_ NS. a) HAADF–STEM–EELS mapping performed across the lateral dimension, and b) EDS of a prepared lamella cut out of a NS by FIB. c–e) XPS Se 3d analysis of binary Bi_2_Te_3_ and Bi_2_Se_3_, and ternary Bi_2_Te_3–*x*_Se_*x*_ NSs. XPS sputtering experiments highlight the change in elemental ratios in NSs having core/shell structure (c,d), and alloyed structure (e) shown on the example of the Se 3d signal. f) Scheme of two ternary materials that differ in their number of crystal phases, i.e., homogeneously alloyed and heterostructured, and g) Raman spectra of binary and ternary NSs acquired with 488 nm laser excitation.

The results from XRD and SEM give insights into the formation of the NSs. The growth in the form of heterostructured Bi_2_Te_2_Se/Bi_2_Te_3_ can be explained by the timely separation of selenium and tellurium precursor decomposition into the reactive Se and Te species. The thickness of the produced heterostructured NSs was investigated using atomic force microscopy (AFM), SEM of free‐standing sheets, and TEM imaging and found to be approximately 50–60 nm (see Figure SI7, Supporting Information). The elemental distribution over the lateral dimension and thickness of the ternary NSs were studied by scanning TEM (STEM). For evaluating thickness‐related elemental distribution, lamellae were cut from Bi_2_Te_2.55_Se_0.45_ NSs by a focused ion beam (FIB). By using high angle annular dark‐field (HAADF)–STEM–EDS as well as electron energy loss spectroscopy (EELS) analyses, we revealed the core/shell structure and thereby the distinct layers of Bi_2_Te_2_Se/Bi_2_Te_3_. The scan across the lateral direction presented in **Figure** **3**a showed a Se‐rich hexagonal NS core, surrounded by a Te‐rich shell. In the FIB‐cut lamella (Figure 3b), the expected layers are visible across the NS thickness (Bi_2_Te_3_/Bi_2_Te_2_Se/Bi_2_Te_3_). The surface of the NSs is richer in oxygen than the inner part, which can be due to oxidation or the influence of bound polyvinylpyrrolidone (PVP) used as a ligand in the synthesis. The STEM images proved the expected heterostructure, which is the first example of a core/shell structure of Bi_2_Te_2_Se/Bi_2_Te_3_ that could be synthesized in an one‐step procedure. More STEM images of flat‐lying sheets were obtained for samples with the compositions of Bi_2_Te_2_Se, Bi_2_Te_2.4_Se_0.6_, and Bi_2_Te_2.7_Se_0.3_. In Bi_2_Te_2.55_Se_0.45_ and Bi_2_Te_2.4_Se_0.6_ samples (Figure 3a and Figure SI8a, Supporting Information), the core/shell structure was detected across the lateral dimension of the NSs. In the case of Bi_2_Te_2.7_Se_0.3_ NSs, clear separated phases were not observed using STEM (Figure SI8b, Supporting Information). The reason is the relatively small amount of Se in the sample, which makes up only 6% of the mole fraction. An estimation of error in EDS measurements ranges up to 5% and for such thin structures below 100 nm the detection is prone to errors. As expected for Bi_2_Te_2_Se, no heterostructure was observed (see Figure SI8c, Supporting Information). The three elements were found homogeneously distributed across the sheet, corroborating with the XRD results that show only reflexes of one crystal phase. It was also found in ternary samples, where the Te:Se ratio was held above 2:1, that thin and holey Bi_2_Te_3_ NSs have evolved sometimes as a side product, but most of them were removed by thorough washing, sonication, and precipitation steps.

Chemical composition of the samples was further studied by X‐ray photoelectron spectroscopy (XPS) combined with sputtering experiments designed to probe the thickness‐dependent elemental distribution qualitatively (Figure 3c–e). Taking Se 3d signal as a reference, we monitored its change during the sputtering. In the heterostructure with the composition Bi_2_Te_2.55_Se_0.45_ selenium was hardly detectable before sputtering (Figure 3c,d), whereas after sputtering a Se peak appears. This is even more pronounced in the annealed sample. These XPS results combined with the images from STEM give a further proof for the core/shell structure with Se‐ and Te‐rich layers (Bi_2_Te_3_/Bi_2_Te_2_Se/Bi_2_Te_3_). The opposite is true for a sample with the composition of Bi_2_Te_2_Se, as expected; the signals in the Se 3d spectrum can be observed before and after several sputtering cycles, showing the homogeneously alloyed structure of these NSs (Figure 3e). Additional results of XPS measurements (Figures SI9 and SI10) and their discussion are given in the Supporting Information. The data obtained from Raman measurements well reflect the findings for the ternary heterostructured NSs and their binary counterparts. In pure Bi_2_Te_3_ and Bi_2_Se_3_, the characteristic *E* ^2^_g_ and *A* ^2^_1g_ modes were observed at frequencies of 103 and 137 cm^−1^, and 132 and 176 cm^−1^, respectively (Figure 3e). This is in good agreement with earlier reports.^[^ ^31^ ^]^ In the heterostructured NSs with a nominal composition of Bi_2_Te_2.4_Se_0.6_ and Bi_2_Te_2.55_Se_0.45_, the frequencies of both Raman modes are not shifted to the values of 106 and 150 cm^−1^ expected for an alloy of the given composition. Instead, the Raman bands of the heterostructured NSs were broadened due to the two crystal phases present in one species, confirming the core/shell structure of the NSs.

### 2.2 Postsynthetic Treatment and Consolidation of the NSs into Nanostructured Solids

**Figure 4:**
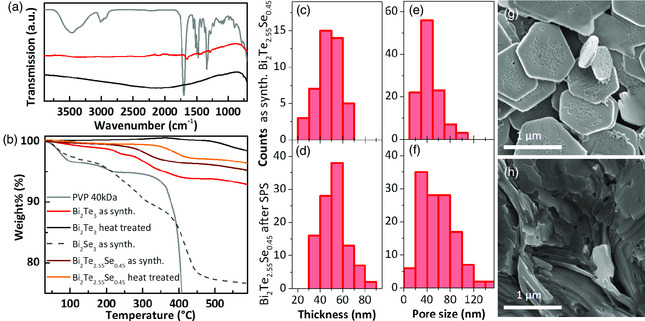
a) ATR‐FTIR spectra of PVP, Bi_2_Te_3_, and Bi_2_Se_3_ NSs before and after the heat treatment. b) TGA curves of Bi_2_Te_3_, Bi_2_Se_3_, and heterostructured Bi_2_Te_2.55_Se_0.45_ NSs before and after the treatment. SEM images of g) as‐synthesized and h) sintered Bi_2_Te_2.55_Se_0.45_ NSs with the corresponding c,d) sheet thickness and e,f) pore size distributions.

The binary and ternary NSs synthesized as described earlier were obtained as dispersions in a low boiling solvent such as *i*PrOH. For a full thermoelectric characterization, which includes measuring transport properties such as thermal conductivity, electrical conductivity, and Seebeck coefficient, the material needs to be compacted. The compaction performed using SPS can have a large influence on the transport properties of the material and is, therefore, discussed in the following section. In addition, as the NSs were synthesized in ethylene glycol in the presence of PVP, an insulating stabilizer, a pretreatment of the nanomaterials to remove organic residues from their surface is necessary to ensure well‐densified structures, in which particles are in close contact. Directly after the synthesis of the NSs, large amounts of excessive PVP, unreacted salts, and high boiling solvent ethylene glycol were removed by several washing steps with low boiling solvents acetone and *i*PrOH. The precipitated NSs were then dried under vacuum overnight to remove the solvents. A powder of Bi_2_Te_3_ NSs after drying (termed: as synthesized) was characterized using attenuated total reflectance Fourier transform infrared (ATR‐FTIR) spectroscopy and compared with the spectrum of pure PVP (**Figure** **4**a). Its results indicate that residuals of PVP still remain in the samples; the polymer is identified in Bi_2_Te_3_ powders by small bands at 1288 cm^−1^ (C—H), 1422 cm^−1^ (C—N), and 1647 cm^−1^ (C=O stretching). Thermogravimetric analysis (TGA) of such annealed Bi_2_Te_3_ NSs sample (30 min at 350 °C) indicated no significant weight loss in the temperature range between 30 and 520 °C, implying that no PVP remains on the surface of these NSs. In the ternary system Bi_2_Te_2.55_Se_0.45_, a small weight reduction was observed at 300 °C. After heat‐treatment weight loss occurred in this sample at temperatures higher than 350 °C, which marks the highest applicable temperature.

Thus, at 350 °C for 30 min annealed samples were used for the compaction by SPS to evaluate their thermoelectric properties. In particular, the nanostructured bulk pellets were produced by two SPS methods: *A*) in graphite (C) dies at 350 °C under an applied uniaxial pressure of 100 MPa, and *B*) in tungsten carbide (WC) dies at 350 °C under 700 MPa. By applying *method A*, we reached densities of the pellets of about 80–83%, compared with the crystallographic theoretical values. Using *method B*, we increased the degree of densification to 87–91%. Similarly low densities were observed for porous nanocomposites made of the Bi_2_Te_2.55_Se_0.45_ holey nanostructures, which can be explained by the morphological peculiarities of these species.^[^ ^32^ ^]^ By SEM imaging of the pelletized samples we observed that the sizes of the sintered sheets did not increase (Figure 4c–h), and thus the fine grain structure was preserved, which is very important for an efficient reduction of the thermal conductivity. Before and after processing, the porous structure of the NSs can be observed with an increase in hole size after the sintering process. The evaluation of the sample's grain thicknesses yielded no significant variation before and after processing (Figure 4c,d), showing that the SPS compaction works without destroying the most important traits of the engineered nanomaterial. In our experiments we found that the applied uniaxial pressure plays a crucial role in the degree of densification.

**Figure 5:**
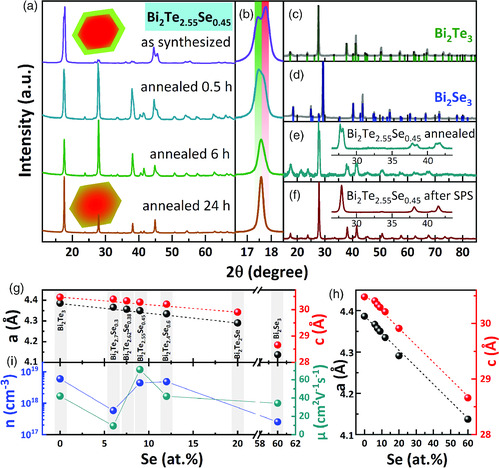
a,b) XRD patterns of Bi_2_Te_2.55_Se_0.45_ NS powders before and after annealing, c–f) XRPD analysis of binary and ternary NSs. Bi_2_Te_2.55_Se_0.45_ NS powders annealed for 30 min, 6 h, and 24 h (a, b). The two crystal phases seemingly merge into an alloyed phase, while a slight asymmetry of the reflexes suggests a persisting crystal phase inhomogeneity. XRPD analyses of Bi_2_Te_3_ (c) and Bi_2_Se_3_ (d) powders with corresponding references, as well as Bi_2_Te_2.55_Se_0.45_ powders after annealing for 30 min (e) and after SPS (f) (*method B*). g,h) The hexagonal unit cell lattice parameters of binary and ternary Bi_2_Te_3–*x*_Se_*x*_ samples after sintering, i) with the corresponding values of the charge carrier concentration and the mobility versus the Se content.

The cylindrical pellets produced using *method B* were 6 mm in diameter and approximately 1 mm thick. The pieces of compacted pellets left after cutting into bars for subsequent thermoelectric characterization (see the next section) were ground into powders for further X‐ray powder diffraction (XRPD) analysis. The difference between XRD and XRPD stems from the sample preparation before the measurement. While for XRD dispersions were drop‐cast on a Si‐wafer, for XRPD powders were immobilized between polymer sheets. Thereby, in XRPD, results are less affected by anisotropy. The XRD results discussed earlier show that Bi_2_Te_3_, Bi_2_Se_3_, and Bi_2_Te_2_Se grew as single‐phase materials with similar crystal structures (R‐3m). Ternary NSs with Te:Se ratios above 2:1 were grown as Bi_2_Te_2_Se/Bi_2_Te_3_ core/shell materials. By annealing at 350 °C it was possible to controllably induce further alloying as can be seen in XRD experiments performed on samples of core/shell Bi_2_Te_2.55_Se_0.45_ NSs (**Figure** **5**a). After 30 min only small changes were observed, but after the SPS treatment the nanomaterials become more homogeneous as seen from XRPD patterns of a powder after sintering (Figure 5e,f), although in all XRPD patterns of SPS‐compacted samples the peaks are broad and asymmetric. This implies a degree of inhomogeneity in the final products. The annealing experiments revealed that after 6 h the (006) reflexes around 17.5° 2*θ* have merged, resulting in a strongly asymmetric signal. This asymmetry can be sign for the evolution of a gradient composition across the NSs. Such a gradient induced by annealing at elevated temperatures was also found in much smaller nanoparticle systems, such as 10 nm CdSe/ZnSe.^[^ ^33^ ^]^ After 24 h the signal became more symmetric, yet a slightly inhomogeneous distribution of the elements across the NSs cannot be excluded. In addition, the corresponding reflexes of Bi_2_Te_3_ and Bi_2_Te_2_Se already laid close to each other. Small differences or gradients between phase compositions in the alloyed NSs were difficult to detect. The features of the obtained nanomaterials will be shown to have a direct influence on their thermoelectric properties. In this regard, the possibility of tuning the NS heterostructure further into a gradient composition presents an additional optimization strategy to improve thermoelectric properties.

The sample bars prepared for the electrical transport measurements were used to determine Hall resistivity, mobility, and carrier concentration. The results from XRPD and Hall measurements are shown in Figure 5g–i. It was found that bulk Bi_2_Te_3—*x*_Se_*x*_ alloys exhibit a small deviation of the lattice parameters from Vegard's law for the compositions with *x* ≥ 1.^[^ ^34^ ^]^ Furthermore, investigations unveiled that the samples with *x* = 0.67–1.45 exhibit a demixing zone between two phases with the same structure, while the sample with *x* = 1 transforms into a single‐phase material with a different and unknown metastable crystal structure.^[^ ^35^ ^]^ The same feature, i.e., an inhomogeneity of the final products, was observed after the synthesis of a similar nanopowder.^[^ ^36^ ^]^ The lattice parameters of the SPS‐sintered samples linearly decrease with the Se content (Figure 5g,h), which is an additional indicator of the formation of ternary alloys with the respective Te:Se ratios. The linear trend until *x* = 1 was also observed in other types of nanoparticles, which were synthesized under different synthetic conditions.^[^ ^11^ ^]^ However, the lattice parameters represent only an average macrostructure, whereas on the atomic scale, apparently, the structure is more complex. This complexity is reflected in the nonlinear trend of the charge carrier concentration and the mobility depending on the composition of the NSs shown in Figure 5i. According to these results, pure Bi_2_Te_3_ has the highest charge carrier concentration among all studied samples, whereas Bi_2_Te_2.55_Se_0.45_ sample exhibits the highest value of the mobility.

### 2.3 Thermoelectric Properties of the Nanostructured Solids

**Figure 6:**
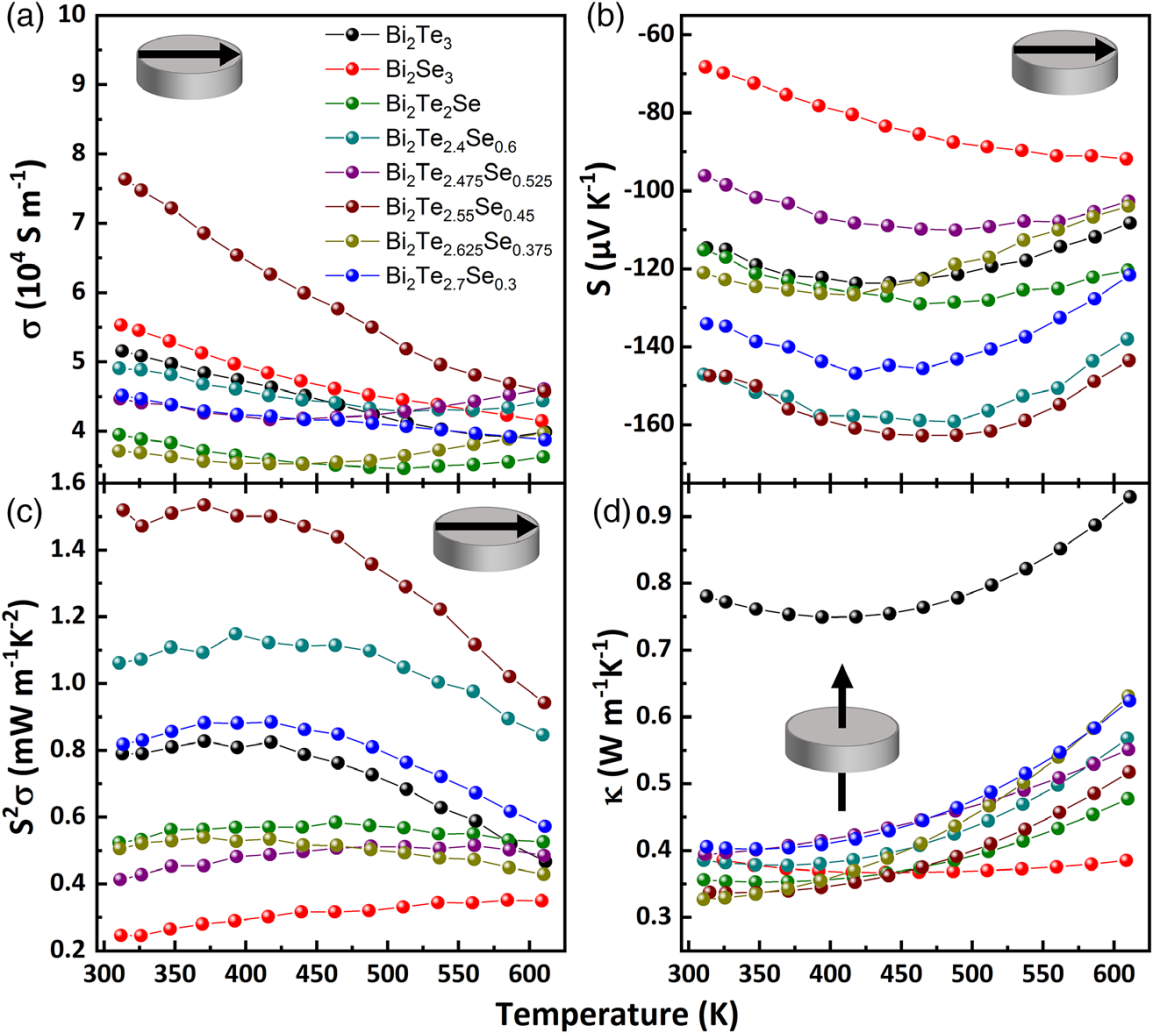
a) Electrical conductivity, b) Seebeck coefficient, c) power factor, and d) thermal conductivity values of Bi_2_Se_3_, Bi_2_Te_3_, and Bi_2_Te_3—*x*_Se_*x*_ samples.

The thermal conductivity in the pellets was determined in pressing direction (cross‐plane) by measuring the thermal diffusivity in an LFA setup. The electrical conductivity and Seebeck coefficient were measured perpendicular to the pressing direction from bars that were cut out of the cylindrical pellets. We investigated samples prepared by *method B* ensuring a dense structure of the solids. As shown in **Figure** **6**a, the electrical conductivities decrease monotonically with rising temperature for all investigated compounds, suggesting that all materials behave as degenerate semiconductors. The binary phases exhibit similar values of the electrical conductivity as the alloyed ones. Admittedly, in the case of the ternary samples no distinct trend in the values is observed depending on the composition (see also Figure SI11, Supporting Information), although some regularities are noticeable. Negative values of the Seebeck coefficient for all samples and Hall resistivity measurements indicate n‐type conduction.

The highest electrical conductivity was measured for the heterostructure with a Te:Se ratio of 85:15 (Bi_2_Te_2.55_Se_0.45_), while the concentration of the charge carriers was comparable with the values of pure Bi_2_Te_3_ and Bi_2_Te_2.4_Se_0.6_ samples (see Figure 5i). At the same time, Bi_2_Te_2.55_Se_0.45_ exhibited the highest value of the electron mobility, which is the reason of its high conductivity. The best values of the Seebeck coefficient were observed in the Bi_2_Te_2.4_Se_0.6_ and Bi_2_Te_2.55_Se_0.45_ samples. As shown in Figure 6b, with increasing temperature the absolute values of the Seebeck coefficient for all samples except Bi_2_Se_3_ pass through maxima and then start to decrease at higher temperature, which is evidence for the bipolar conduction behavior, typical for narrow band gap semiconductors or semimetals. With increasing Se content, the electrical conductivity and the Seebeck coefficient values display opposite trends leading to an enhancement of the power factor. The Bi_2_Te_2.55_Se_0.45_ sample showed the highest power factor of 1.58 mW m^−1^ K^−2^ at 370 K (Figure 6c). This result is unexpected, as the composition Bi_2_Te_2.7_Se_0.3_ is outperformed in our experiments. To this date, the highest *zT* of 1.2–1.31 was measured for compacted Bi_2_Te_2.7_Se_0.3_ NSs, reaching similar power factors under comparable sintering conditions.^[^ ^11^, ^19^, ^31^, ^37^ ^]^ In an earlier publication from 2013, Bi_2_Te_2.55_Se_0.45_ composition produced from mixtures of Bi_2_Te_3_ and Bi_2_Se_3_ NSs achieved a power factor of 1.2 mW m^−1^ K^−2^ (400 K) with low thermal conductivity resulting in a *zT* of 0.71. Later, in 2017, a *zT* of 1.18 was achieved by Xu et al. reducing the thermal conductivity while maintaining the power factor in sintered hollow Bi_2_Te_2.55_Se_0.45_ nanostructures.^[^ ^32^ ^]^

The thermal conductivities of all samples sintered using *method B* were measured parallel to the pressing direction (cross‐plane), and exhibited considerably reduced values compared with the corresponding bulk counterparts^[^ ^38^, ^39^ ^]^ and early‐reported nanostructured materials.^[^ ^11^, ^37^, ^40^, ^41^ ^]^ The thermal conductivity was especially low for the ternary compounds and Bi_2_Se_3_, *κ* drops with increasing Se content reaching the lowest values in the case of the heterostructured Bi_2_Te_2.55_Se_0.45_ (Figure 6d). The low thermal conductivity in the Se‐containing NSs can be explained by dislocations resulting in wide‐frequency phonon scattering.^[^ ^11^ ^]^ Second, strong reduction of the thermal conductivity is the result of efficient nanostructuring. Here, it should be mentioned that not only grain boundaries between NSs act as efficient scattering centers, but also the repeating core/shell structure inside the pellet serves this purpose. The increase in thermal conductivity with temperature occurs for every sample apart from Bi_2_Se_3_, similar to observations for Seebeck coefficient. This effect can be explained by the influence of bipolar conduction.

**Figure 7:**
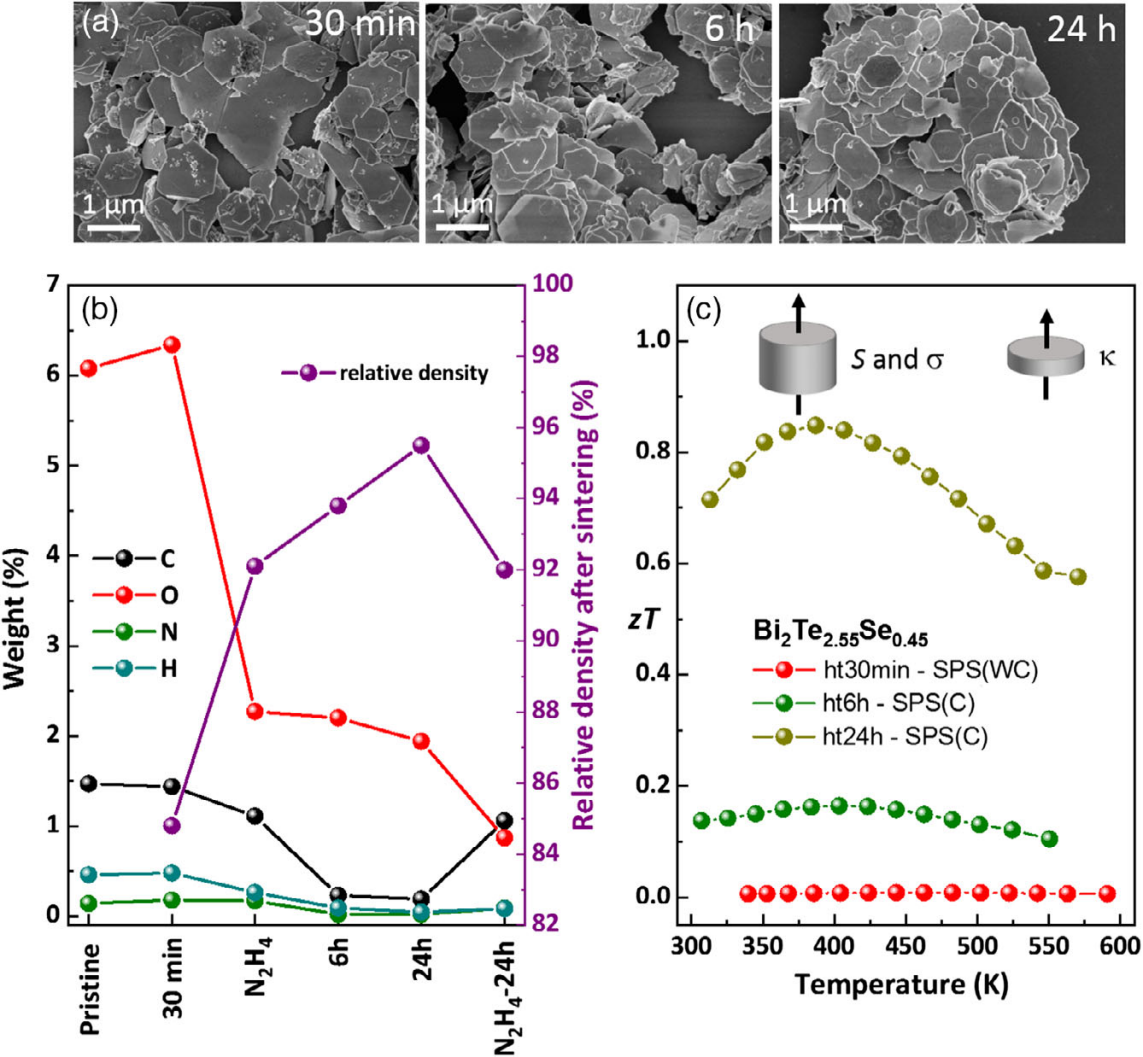
Comparison of the light element content in dried, annealed, and/or chemically treated Bi_2_Te_2.55_Se_0.45_ NS powders. a) SEM images of NSs annealed at 350 °C for 30 min, 6, and 24 h. b) Light element analysis of annealed or chemically treated Bi_2_Te_2.55_Se_0.45_ NS powders, and the resulting relative density after SPS (*method A*). c) Thermoelectric figure of merit of Bi_2_Te_2.55_Se_0.45_ NSs determined from Seebeck coefficient, electrical, and thermal conductivity measured cross‐plane as indicated.

The sample Bi_2_Te_2.55_Se_0.45_ showed the best power factor in our measurements and a low thermal conductivity and, therefore, its thermoelectric properties were investigated in more detail. The pellets produced in this work were characterized by SEM, revealing mostly ordered stacking of the NSs inside the pellet (see Figure SI12, Supporting Information). This inner arrangement is a strong indicator of anisotropy of the materials. Both ordered and disordered lamellar structures with small angle grain boundaries, formed by face‐to‐face packed NSs, can be observed, which are beneficial for an efficient nanostructured thermoelectric, as they introduce scattering sites to reduce the lattice thermal conductivity. This was confirmed also for the Bi_2_Te_2.55_Se_0.45_ sample cut with a razor blade (Figure SI13, Supporting Information). Recently, isotropy was demonstrated in similar 2D nanomaterials sintered into thick pellets followed by cutting them perpendicular (in‐plane) and parallel (cross‐plane) to the uniaxial pressure direction with subsequent thermoelectric characterization.^[^ ^11^, ^42^ ^]^ In these studies nearly identical values of the parameters in both directions were measured, revealing no favorable direction of the electrical and thermal transport. In contrast to these results, in the similar system of alloyed Bi_2_Te_3—*x*_Se_*x*_ with addition of tellurium nanorods, strong difference between in‐plane and cross‐plane directions was found.^[^ ^19^ ^]^ These studies emphasize the importance of the measurement geometry, i.e., that all transport measurements (thermal and electric) are aligned in the same direction. For this purpose, we have prepared thicker pellets of Bi_2_Te_2.55_Se_0.45_ sample (3 mm height), which were measured in parallel to the pressing direction using LSR‐3 device for determination of the Seebeck coefficient and electrical conductivity. These measurements revealed a strong deviation from the in‐plane characterization with electrical conductivities being three magnitudes smaller. We assign the origin of this low electrical conductivity to the presence of residuals of PVP that were not observed by TGA and FTIR. By characterization of light element (C, O, N, H) content in differently annealed or hydrazine‐treated powders, we found that this value can be drastically reduced with prolonged annealing times up to 24 h (**Figure** **7**b). The shape of the NSs after annealing changed from regular hexagons, while the sheet‐like morphology was preserved (see Figure 7a). In addition, higher densities were achieved after longer annealing or hydrazine treatment even by using *method A*. Powders annealed for 6 and 24 h and sintered by *method A* were compacted into thick and thin pellets to measure cross‐plane electrical conductivity, thermal conductivity, and Seebeck coefficient. The measurements showed that the electrical conductivity was improved due to the annealing and removal of light elements, while thermal conductivity was similar in each sample (see Figure SI13, Supporting Information). By this prolonged annealing, *zT* could be increased from nearly 0 to 0.85, as shown in Figure 7c.

The synthesized Bi_2_Te_2.55_Se_0.45_ NSs are similar to the Bi_2_Te_2.7_Se_0.3_/Bi_2_Te_3_ NSs produced by Li et al., with *zT* measured cross‐plane to be around 1.17.^[^ ^29^ ^]^ Both materials exhibit equally low thermal conductivity as a result of the core/shell structure, but our material shows stronger dependence of heat transport on the temperature and slightly lower power factors. The power factors achieved for cross‐plane direction are only about half of that measured before in‐plane. In fact, well‐oriented polycrystalline materials have been shown before to exhibit higher power factors in in‐plane direction (along the ab plane) compared with cross‐plane direction.^[^ ^19^, ^43^, ^44^ ^]^ This can be explained by the higher electrical conductivity, thereby higher *κ*_el_ as well as reduced number of grain boundaries along the pellet. Means to decrease lattice thermal conductivity along in‐plane direction are, therefore, supposed to have a large impact on overall *zT* value. The Bi_2_Te_2_Se/Bi_2_Te_3_ samples fabricated in our work are comparable to the study of colloidally synthesized multishell NSs (Bi_2_Se_3_)_*m*_/(Bi_2_Te_3_)_*n*_ that demonstrated a peak *zT* of ≈0.71 measured in‐plane, having very low thermal conductivities and good power factors.^[^ ^28^ ^]^ The core/shell nanomaterials possess additional structuring alongside the lateral direction, reducing the thermal conductivity while maintaining high electrical conductivities. In comparison to the study of (Bi_2_Se_3_)_*m*_/(Bi_2_Te_3_)_*n*_, power factors achieved for in‐plane direction in this work are about 50% higher in the Bi_2_Te_2.55_Se_0.45_ sample. Proving reduced thermal conductivity in the core/shell structure by in‐plane measurements is the last challenge discussed in this work. For this, we have determined the thermal conductivity of Bi_2_Te_3_, Bi_2_Se_3_, and Bi_2_Te_2.55_Se_0.45_ samples using an in‐plane measurement sample holder for LFA, while we obtained values of Seebeck coefficient and electrical conductivity by LSR‐3 setup for cylinder in‐plane measurements. The samples were annealed for 24 h (or treated with hydrazine in ethanol to remove the ligands) and sintered using *method A*.

**Figure 8:**
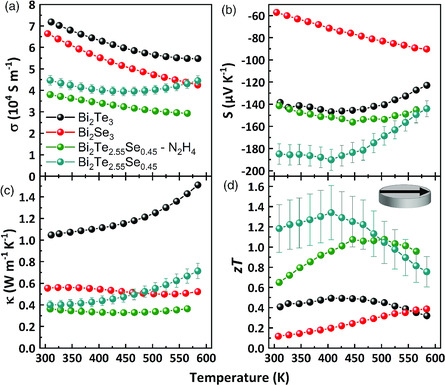
Full in‐plane thermoelectric characterization of Bi_2_Te_3_, Bi_2_Se_3_, and Bi_2_Te_2.55_Se_0.45_ NSs sintered using *method A*. Powders were annealed for 24 h at 350 °C or treated with diluted hydrazine in ethanol before SPS. a) Electrical conductivity, b) Seebeck coefficient, c) thermal conductivity, and d) *zT* values. Error bars represent the measurement errors of the best sample, which is associated with the procedure to determine *zT*.

Pure Bi_2_Te_3_ NSs sample processed using this protocol exhibited high Seebeck coefficients and high electrical conductivity, with a power factor reaching approximately 1.4 mW m^−1^ K^−2^. In comparison with the Se‐containing samples, it suffers from high thermal conductivity which is more than twice that of pure Bi_2_Se_3_. Consequently, the resulting *zT* of 0.49 for pure Bi_2_Te_3_ is relatively low. In Bi_2_Se_3_, the high electrical conductivity and reduced thermal conductivity slightly above 0.5 W m^−1^ K^−1^ are outbalanced by a small value of *S* between −60 and −90 μV K^−1^ (300–584 K). Therefore, a poor overall *zT* is obtained having a peak value of 0.39 at 584 K. The core/shell NSs processed by annealing for 24 h or chemical treatment both exhibited reduced values for in‐plane *κ*. The same arguments for low *κ* that were discussed for cross‐plane measured pellets (*method B*) are also true for these materials. In comparison with Bi_2_Te_2.55_Se_0.45_ annealed for 24 h, the hydrazine‐treated sample possessed a less pronounced temperature dependence of *κ*. This behavior can be explained by the structural differences inside the pellets. The hydrazine‐treated sample was not annealed; therefore, no alloying before SPS occurred. Consequently, the Bi_2_Te_2_Se/Bi_2_Te_3_ heterostructure was preserved better, introducing many grain boundaries and having a pronounced core/shell substructure as shown by XRD characterization (see Figure SI16, Supporting Information). Lattice thermal conductivities for the samples shown in **Figure** **8** were estimated using Wiedemann–Franz law (see Figure SI17, Supporting Information). The extremely low values for *κ*–*κ*_el_ between approximately 0.15 and 0.25 W m^−1^ K^−1^ in our best sample are the result of an effective nanostructuring (a large number of introduced grain boundaries and holes) and heterostructuring combined with the effects of Se‐doping. These low lattice thermal conductivity values are comparable to low lattice thermal conductivities obtained for heterostructure Bi_2_Te_2.7_Se_0.3_/Bi_2_Te_3_ by Li et al.,^[^ ^29^ ^]^ and are slightly lower than the theoretically calculated minimum lattice thermal conductivity (0.18 W m^−1^ K^−1^) for randomly ordered, fully dense Bi_2_Te_3_.^[^ ^45^ ^]^ The rise of *κ* with increased temperature in the annealed sample can be explained by bipolar conductivity, which is also the reason for decreasing Seebeck coefficient after passing the maximum value. Materials with a larger bandgap are less affected by minority carriers; therefore, the presence of a larger bandgap material such as Bi_2_Te_2_Se in the pellet suppresses bipolar conduction effectively. Comparing the two pellets, a disadvantage of the strategy to preserve Bi_2_Te_2_Se/Bi_2_Te_3_ becomes apparent. The electrical conductivity suffers from the presence of different phases where not only the phonons but also electrons can be scattered. By this, the power factor reached only 0.8 mW m^−1^ K^−2^ in the hydrazine treated sample, while in the annealed sample values of 1.45–1.5 mW^−1^ K^−2^ were achieved. Nevertheless, a high *zT* of 1.08 was achieved in hydrazine‐treated samples after sintering. The elemental distribution of Se in the Bi_2_Te_2.55_Se_0.45_ NSs after 24 h annealing is most likely to change from the core/shell to a gradient one. This may be concluded from the observation of merging reflexes in XRD patterns of NSs after annealing (see Figure SI16, Supporting Information).

An inhomogeneously alloyed structure explains the low in‐plane thermal conductivities in comparison with other publications.^[^ ^11^, ^19^ ^]^ A gradient of Se‐content across the lateral dimension is capable of strongly mitigating phonon transport through scattering point defects and lattice distortions.^[^ ^37^, ^46^ ^]^ In addition, the influence of the holey structure of the NSs cannot be excluded as a reason for reduced overall thermal conductivity and improved *zT*, as discussed for mesoporous and holey Bi_2_Te_3_,^[^ ^47^, ^48^ ^]^ and other materials.^[^ ^49^ ^]^ SEM images revealed that the nanosized holes were preserved in the Bi_2_Te_2.55_Se_0.45_ samples even after sintering (Figure SI18, Supporting Information). As a result, high *zT* value of 1.34 at 400 K was achieved. This is the highest value reached so far for the n‐type Bi_2_Te_3—*x*_Se_*x*_ system owing to the delicate nanostructure engineering in the compacted material, preserving favorable gradient structure of Bi_2_Te_2_Se/Bi_2_Te_3_ NSs. In addition, in contrast to other published bismuth telluride selenide‐based thermoelectric materials mostly having an elemental composition of Bi_2_Te_2.7_Se_0.3_, 5% less of the expensive tellurium precursor is needed to reach this high *zT* value.

## 3 Conclusions

In this work, a facile synthesis of novel material Bi_2_Te_2_Se/Bi_2_Te_3_ core/shell NSs requiring a simple one‐pot procedure and no use of hazardous chemicals is described. Thorough characterization of their morphology, crystal structure, and composition was performed to elucidate their formation mechanism and confirm the core/shell structure and single‐crystalline arrangement of each phase. The produced NSs are uniform in size, show nanosized holes, and possess favorable features such as a core/shell structure dividing the 50–60 nm‐thick sheets into individual sublayers. Nanopowders annealed at 350 °C for 30 min were sintered using a high pressure SPS method, resulting in nearly 90% relative density. Electrical transport and Hall measurements (in‐plane) give an overview of the properties of binary and ternary Bi_2_Te_3—*x*_Se_*x*_ NSs, highlighting the best power factor for the composition of Bi_2_Te_2.55_Se_0.45_. The thermal conductivity of the materials measured cross‐plane shows extremely low values for all Se‐rich NSs as a result of efficient nanostructuring. The composition Bi_2_Te_2.55_Se_0.45_ that has shown best performances in thermoelectric measurements and a distinct core/shell structure is discussed thoroughly focusing on its in‐plane and cross‐plane direction characterization. It is found that the annealing time must be increased to 24 h to ensure satisfactory electrical conductivity by reducing the content of PVP used as a ligand in the synthesis. By this annealing more complete removal of organics can be achieved than through an established routine based on washing with toxic hydrazine. Consequently, even a low‐pressure sintering method can be used for high‐density compaction. A figure of merit of 0.85 is achieved in cross‐plane measurements. In‐plane thermal conductivities were measured using an in‐plane LFA sample holder, revealing low thermal conductivity and high power factors as a result of the unique substructuring. Thereby, a very high *zT* over 1.34 at 400 K is achieved for 24 h annealed Bi_2_Te_2_Se/Bi_2_Te_3_ core/shell NSs (having an average *zT* value of 1.23 between 300 and 500 K).

## Conflict of Interest

The authors declare no conflict of interest.

## Supporting information

Supplementary Material
