# Supplementary material for: Heterostructured Bismuth Telluride Selenide Nanosheets for Enhanced Thermoelectric Performance
Source: Small Sci. 2020 Oct 25;1(1):2000021. doi: 10.1002/smsc.202000021 (PMC11935927; doi:10.1002/smsc.202000021)
Supplement: Supplementary file 1 — Supplementary Material [file SMSC-1-2000021-s001.pdf]

Supporting Information

Heterostructured Bismuth Telluride Selenide Nanosheets for Enhanced Thermoelectric Performance

Christoph Bauer,[1,†] Igor Veremchuk,[2,†] Christof Kunze,[1] Albrecht Benad,[1] Volodymyr M. Dzhagan,[3,4] Danny Haubold,[1] Darius Pohl,[5] Gabi Schierning,[6] Kornelius Nielsch,[6-8] Vladimir Lesnyak[1]*, Alexander Eychmüller[1]

[1]Physical Chemistry, TU Dresden, Bergstr. 66b, 01062 Dresden, Germany

[2]Max Planck Institute of Chemical Physics for Solids, Nöthnitzer Str. 40, 01187 Dresden, Germany

[3]Semiconductor Physics, Chemnitz University of Technology, Reichenhainer Str. 70, 09126 Chemnitz, Germany

[4]Institute of Semiconductor Physics, Nat. Acad. of Sciences of Ukraine, Nauky av. 45, 03028 Kyiv, Ukraine

[5]Dresden Center for Nanoanalysis, TU Dresden, Helmholtzstraße 18, 01069 Dresden, Germany

[6]Leibniz Institute for Solid State and Materials Research Dresden, Helmholtzstraße 20, 01069 Dresden, Germany

[7]Institute of Applied Physics, TU Dresden, Nöthnitzer Str. 61, 01187 Dresden

[8]Institute of Materials Science, TU Dresden, Helmholtzstr. 7, 01069 Dresden

[†]These authors contributed equally to the work.

EXPERIMENTAL SECTION

Materials. Ethylene glycol (EG, ≥99.5%) and acetone (*p.A.*) were purchased from Honeywell Riedel-de Haen. Na_2SeO_3 (99%), Na_2TeO_3 (99%), $Bi(NO_3)_3\cdot5H_2O$ (99.999%), polyvinylpyrrolidone (PVP, M_w=40 kDa), and hydrazine-monohydrate (98%) were purchased from Sigma-Aldrich. *Iso*-Propanol (*i*PrOH*, p.A.*) was purchased from Fisher. Sodium hydroxide (NaOH, 99%) was purchased from Grüssing and absolute ethanol from VWR.

SYNTHETIC PROCEDURES

Synthesis of bismuth telluride, bismuth selenide and their alloyed/heterostructured NSs.
The syntheses of pure Bi_2Te_3 and Bi_2Se_3 NSs were adopted from a recipe published by Lin *et al.*[1] To obtain significant amounts of the materials for thermoelectric characterization, the original recipes were scaled up to 15-fold.

For the **Bi_2Te_3 NS synthesis**, first a solution of 2.4 g (60 mmol) of NaOH in 100 mL of EG was prepared by stirring the mixture at 100°C. The resulting solution was added to 1.455 g (3 mmol) of $Bi(NO_3)_3\cdot5H_2O$, 0.9825 g (4.5 mmol) of Na_2TeO_3, 3.3345 g (30 mmol) of PVP, and 50 mL of EG in a 250 mL three-neck flask with an attached condenser. The solution was rapidly heated to 190°C with a heating mantle under vigorous stirring. After reaching the desired temperature the reaction was left stirring for 3 h. Thereafter, the mixture was cooled down to room temperature. Purification of the NSs was done with iPrOH/acetone (1:1-vol.) mixture. 30 mL of the crude solution was combined with 20 mL of the mixture and subsequently centrifuged at

5,000 rcf for 10 min. The supernatant was discarded, and the precipitate was redispersed in 10 mL of iPrOH with the help of an ultrasonic bath. 10 mL of acetone were added, and the suspension was centrifuged for 5 min. The last two steps were repeated three times. The obtained precipitate was dried in vacuum at room temperature overnight.

For the **synthesis of Bi_2Se_3 NSs**, 1.455 g (3 mmol) of $Bi(NO_3)_3{\cdot}5H_2O$, 0.7785 g (4.5 mmol) of Na_2SeO_3, 3.3345 g (30 mmol) of PVP, and 150 mL of EG were mixed in a 250 mL three-neck flask with condenser attached. The solution was stirred for 1 h before heating to 190°C. The reaction time at this temperature was set to 2.5 h. The product NSs were purified analogously to Bi_2Te_3 NSs.

Heterostructured NSs were synthesized using a combined procedure. In this case, we varied the tellurium to selenium precursor atomic ratios from 95/5 to 5/95 and added 2.4 g of NaOH analogously to the synthesis of pure bismuth telluride NSs. The reaction time was kept 3 h. For example, to synthesize heterostructured $Bi_2Te_{2.55}Se_{0.45}$ NSs, first, a solution of 2.4 g (60 mmol) of NaOH in 100 mL of EG was prepared by heating and stirring at 100°C. This solution was mixed with 1.455 g (3 mmol) of $Bi(NO_3)_3{\cdot}5H_2O$, 0.8475 g (3.825 mmol) of Na_2TeO_3, 0.1168 g (0.675 mmol) of Na_2SeO_3, 3.3345 g (30 mmol) of PVP, and 50 mL of EG in a three-neck round-bottom flask with an attached condenser. The solution was stirred for 1 h before heating to 190°C with a following reaction time of 3 h at this temperature. The reaction was quenched by removing the heating mantle and the produced NSs were purified as described above.

POSTSYNTHETIC TREATMENT

Before spark plasma sintering (SPS) was conducted, residual solvents and organic surfactant had to be removed from the NSs. Therefore, dried powders were annealed or/and chemically treated using hydrazine monohydrate solution.

Thermal annealing of dried powders was performed in Carbolite EHA 12/300 tube furnace under a nitrogen flow for 30 min (or 2 h, 6 h, 24 h) at 350°C. Powder was loaded in a porcelain weighing boat, the top was carefully sealed with Al-foil which was punctured using a needle. The weighing boats were placed in the middle of a quartz tube that was subsequently positioned inside the tube furnace and purged with nitrogen for 10 min to remove air. Then the temperature was raised to 350°C using a gradient of 5°C/min.

Chemical stripping of residual ligands from dried powders was performed using a 20/80 mixture of hydrazine monohydrate and absolute ethanol. About 100 mL of this mix was added (split in three portions) per 1.5 g powder. The dry powders were dispersed in the mix by ultrasonication for 5 min, the liquid was discarded after precipitation (centrifuge 5,000 rcf for 5 min), the washing was repeated with the hydrazine solution twice. Thereafter, the precipitate was dispersed with consequent centrifugation (5,000 rcf for 5 min) firstly three times using EtOH, and secondly three times using acetone. Eventually, the powder was dried overnight in a desiccator connected to a vacuum oil pump for compaction or for further annealing.

SPS was performed using a 515 ET Sinter Lab (Fuji Electronic Industrial Co. Ltd.). SPS apparatus in an argon-filled glovebox was used for the compaction of annealed and/ or chemically treated binary Bi_2Te_3, Bi_2Se_3 NSs, as well as ternary alloyed and heterostructured $Bi_2Te_{3-x}Se_x$ NSs into dense cylindrical pellets. Powder samples were ground in an agate mortar and filled inside the Ta-foil-lined die, sealed with the punches and transferred in the SPS glovebox. The amount for the compaction depended on the used die, and the desired height of

the pellet. Two different compaction methods were applied, **method *A***: 350°C for 10 min, 100 MPa, 8 mm diameter graphite (C) dies; **method *B***: 350°C for 10 min, 700 MPa, 6 mm tungsten carbide (WC) dies.

CHARACTERIZATION

The morphology as well as the elemental content of all nanomaterials were analyzed by ***scanning electron microscopy*** (SEM) on a SU8000 SE Microscope (Hitachi) operating at 1–10 kV, depending on the sample, equipped with an ***energy dispersive x-ray spectroscopy*** (EDS) setup operating at 20 kV, or on a DSM 982 Gemini (Zeiss). Samples from liquid dispersions were prepared by drop-casting 3-4 µL of a purified dispersion on a Si-wafer with subsequent drying under ambient atmosphere. Pellets after sintering were prepared for SEM on carbon adhesive disks. For this, the pellets were typically broken by force.

High-angle annular dark-field scanning transmission electron microscopy (HAADF-STEM) imaging and element mapping based on ***EDS*** were performed on a Talos F200X microscope equipped with an X-FEG electron source S11 and a Super-X EDS detector system (FEI), operated at 200 kV. ***High resolution-STEM*** and ***electron energy loss spectrometry*** (EELS) were performed on a Titan^3 80-300 (FEI) image and probe corrected microscope operated at 300 kV.

Dual-beam focused ion beam (FIB) FEI Helios 660 was employed for the preparation of $Bi_2Te_{2.55}Se_{0.45}$ single lamellae for STEM measurements. NSs were drop-cast on a Si-wafer. A thin protective carbon coating, and subsequently a Pt-film were deposited using FIB. Thereafter, lamellae were prepared by using Ga-ion FIB with an acceleration voltage of 30 kV.

Atomic force microscopy (AFM) measurements were performed in tapping mode on a Dimension 3100 atomic force microscope on $Bi_2Te_{3-x}Se_x$ NSs.

Fourier transform infrared (FTIR) spectra were acquired with a Nicolet 8700 attenuated total reflectance (ATR) spectrometer (Thermo Scientific). NS powders were softly pressed on the ATR crystal with subsequent drying. Solutions of EG mixed with PVP were deposited directly on the ATR crystal.

Inductively coupled plasma optical emission spectroscopy (ICP-OES) was done on an OPTIMA 7000DV (PerkinElmer) instrument. Samples were prepared by decomposing approximately 2 mg of the corresponding NS-powder with 0.4 mL of concentrated HNO_3 and diluting with 9.6 mL of Milli-Q water.

Elemental chemical analysis for light elements (LEA) was performed on ground powder samples (~30 mg) by using the carrier-gas hot-extraction technique (LECO C200 setup for carbon, and LECO TCH600 setup for oxygen, nitrogen, and hydrogen).

Thermogravimetric analysis (TGA) was performed on a TGA/DSC 1 STAR® System (Mettler Toledo). Samples were filled in an aluminum crucible, and were heated under argon atmosphere from room temperature with a gradient of 4°C min^{-1} to 600°C.

Crystal structure characterization was carried out by using ***powder x-ray diffraction*** (PXRD, Huber G670 camera, Cu $K_{\alpha1}$, λ=1.54056 Å, $\Delta 2\theta$ = 0.005°) analysis. Data were collected at room temperature by the Guinier technique by adding small amounts of LaB_6 (NIST, 660a) as an internal standard. An experimental position of each reflection was corrected by comparison with the standard and lattice parameters were calculated from

least-squares refinement using the WinCSD software.[2] ***X-ray diffraction*** (XRD) analysis of the NS samples was performed on a D2 Phaser (Bruker) using a Si wafer onto which the nanomaterial dispersions were drop-cast and subsequently dried. The diffraction pattern references were obtained from the ICDD database. The ICSD database and Diamond software was used to depict the crystal structures of bismuth tellurides and selenides.

X-ray photoelectron spectroscopy (XPS) measurements of $Bi_2Te_{3-x}Se_x$ NSs were performed with an ESCALAB 250Xi XPS Microprobe (Thermo Scientific) equipped with a monochromatized Al K_α X-ray source ($h\nu$ = 1486.6 eV). The survey and high-resolution spectra were acquired at a bandpass energy of 200 eV and 20 eV, respectively. The binding energy was calibrated with respect to the C 1*s* peak at 284.6 eV. The Avantage (Thermo Scientific) software was used for the XPS spectra analysis and the calculation of the atomic composition of the samples. For XPS measurements the NS dispersions were drop-cast on a Si wafer and dried in vacuum to avoid oxidation. XPS spectra were acquired from as-deposited samples as well as after sputtering the surface (the top NSs layers) with 2 keV Ar-ions for 30 s. Up to three consecutive/alternate sputtering and XPS measurement cycles were performed on each sample.

Raman spectra of $Bi_2Te_{3-x}Se_x$ NSs were collected by exciting samples with the 488 nm diode pumped solid state (DPSS) laser (Sapphire, Coherent) and registered with a spectral resolution of about 3 cm^{-1} using a LabRam HR800 micro-Raman system. For the Raman measurements, the samples were prepared by drop-casting a thin NS film on a pre-cleaned Si substrate (similar to the XPS analysis). The incident laser power was 0.01 mW, in order to avoid sample heating under the microscope objective (50×).

The **densities *ρ* of the samples after SPS** were determined employing an immersion technique (ARCHIMEDES' principle) with ethanol as the medium (equation 1).

$$\rho = \frac{m_{\mathrm{in\ air}} \cdot \rho_{\mathrm{EtOH}}}{m_{\mathrm{in\ air}} - m_{\mathrm{in\ EtOH}}} \quad (1)$$

With $m_{\mathrm{in\ air}}$ being the measured mass of the solid in air, ρ_{EtOH} as the density of ethanol at the measurement temperature, and $m_{\mathrm{in\ EtOH}}$ is the mass of the solid immersed in ethanol.

Laser flash analysis (LFA) was performed in a ***classical setup*** for ***cross-plane direction*** using LFA 457 MicroFlash from Netzsch with a HgCdTe detector. The thermal conductivity was characterized in low-pressure He-atmosphere in a temperature range from 298 up to 623 K (step size of 20 K) by determining the thermal diffusivity λ. The SPS compacted cylindrical pellets with diameters of 6 mm or 8 mm, and thickness of ca. 1 mm were used to measure the cross-plane thermal diffusivity λ under helium atmosphere, with a lowered pressure. The samples were coated with graphite by spraying. The thermal conductivity κ was calculated from the following equation:

$$\kappa = \lambda \cdot \rho \cdot c_p \quad (2)$$

The specific heat capacities c_p were calculated for all $Bi_2Te_{3-x}Se_x$ NSs using literature reference values reported by HONG *et al.*[3]

$$c_p(\mathrm{Bi_2Te_3}) = 108.06 + 5.53 \cdot 10^{-2}\ T\ (\mathrm{J\ K^{-1}\ mol^{-1}}) \quad (3)$$

$$c_p(\mathrm{Bi_2Se_3}) = 118.61 + 1.92 \cdot 10^{-2}\ T\ (\mathrm{J\ K^{-1}\ mol^{-1}}) \quad (4)$$

For ternary compositions, c_p was estimated combining both equations, e.g., for $Bi_2Te_{2.55}Se_{0.45}$ with the ratio of Te:Se = 85/15, it follows:

$$c_p(\mathrm{Bi_2Te_{2.55}Se_{0.45}}) = 0.85 \cdot c_p(\mathrm{Bi_2Te_3}) + 0.15 \cdot c_p(\mathrm{Bi_2Te_3}) \quad (5)$$

Thermal conductivity in ***in-plane direction*** of 5 mm diameter cylinders with a thickness below 0.75 mm was measured on an LFA 1000 (Linseis) instrument with a Ta-based thermocouple of type-C. Only measurement results that could be fitted with a quality above 99% were used to extract thermal conductivities in this work. A sample holder for in-plane measurements for LFA 1000 system is shown in Figure SI 1.

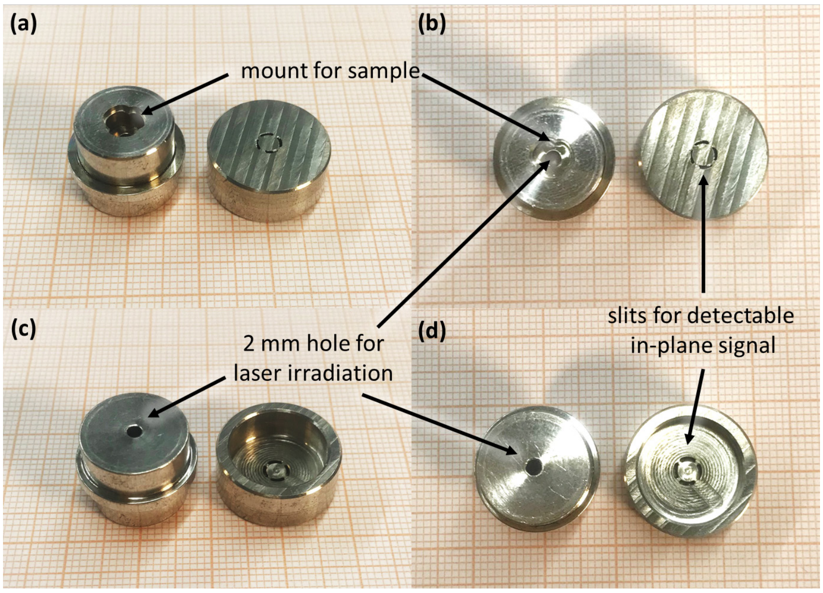

Figure SI 1. Sample holder for LFA in-plane measurements. Photographs (a) and (b) show the top view of the lower part and lid, (c) and (d) show the bottom view of the lower part and lid. The inner circle diameter between slits is 4 mm, slit width of 0.5 mm, and a diameter of the hole in the lower part of 2 mm.

To reduce the size of pellets for this measurement we used a metallographic sample polishing machine (Struers, LaboPol21) rotating 300 rpm with an attached polishing paper (grit 2000). The samples were polished to the desired size and thickness carefully. Sample pellets were fixed on the end of an approx. 20 cm long steel rod using crystalbond (Figure SI 2). The samples were removed from the steel rod by softly heating the steel close to the tip where the sample was attached. Residuals of crystalbond were removed by acetone.

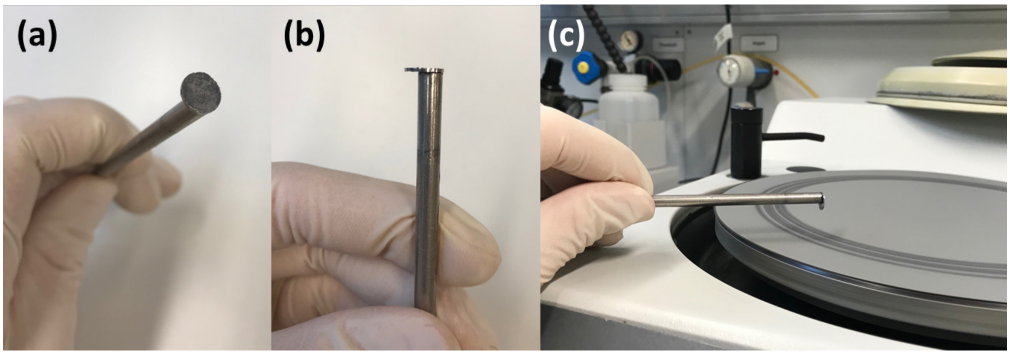

Figure SI 2. Images of SPS pellet (8 mm) attached to a steel rod by crystalbond (a, b), the sample on the steel rod is carefully moved to the rotating polishing paper c).

The measurements of ***electrical conductivity*** σ and ***SEEBECK coefficient*** *S* of bismuth chalcogenide-based samples were performed simultaneously applying a ***direct current four probe method*** and a ***differential***

method, respectively, on a ZEM-3 apparatus (Ulvac-Riko), or on LSR-3 (Linseis) in a temperature range of 298–623 K (step size of 20 K) in He-atmosphere with +0.1 bar overpressure. In ZEM-3 apparatus, sample bars (6×1×1 mm) cut out of the pellets after LFA characterization were measured, thereby obtaining ***in-plane*** *S* and σ. The LSR-3 system can be used to measure in-plane and cross-plane *S* and σ for cylindrical pellets (images of the sample holders are shown in Figure SI 3).

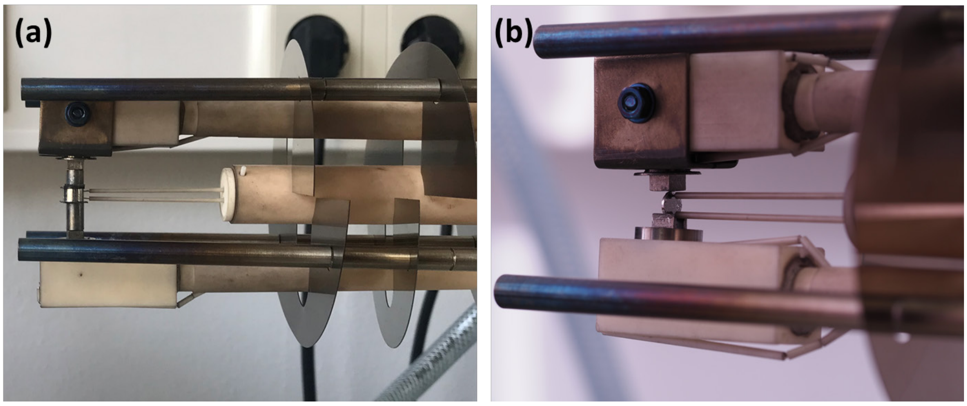

Figure SI 3. Setup of cross-plane (a), and in-plane (b) characterization in LSR-3 system using cylindrical samples to measure electrical conductivity and SEEBECK coefficient. The measurement setup in ZEM-3 would require a standing cuboid bar with length of 6 mm or more.

The cylindrical sample with a height of ca. 3 mm seen in Figure SI 3a can be tilted and measured as seen in (b) but it is not possible to use a thin cylinder to obtain cross-plane data, as the contacts of the thermocouple need a minimum distance of 2.7 mm. The characterization of the SEEBECK coefficient using LSR-3 and ZEM-3 represents a relative measurement method. The thermocouples are made from platinum. *Note:* According to the data from the manufacturers, the estimated measurement errors for $s(\kappa)$, $s(\sigma)$, $s(S)$, and $s(zT)$ are equal to 10%, 5%, 5% and 20%, respectively.

After cross-plane LFA measurements the samples were cut into bars (6×1×1 mm) to measure ***HALL effect*** with a six-point alternating current method in a ***physical property measurement system*** (PPMS, Quantum Design, 5–350 K), with magnetic fields of up to 9 T. The carrier concentration *p* and the mobility μ were calculated as follows:

$$p = (e \cdot R_{\mathrm{H}})^{-1} \quad (6)$$

$$\mu = \sigma \cdot (\rho \cdot e)^{-1} \quad (7)$$

where R_H is the HALL-coefficient ($cm^3\ C^{-1}$) and *e* is the electron charge that equals $1.602 \cdot 10^{-19}$ C.

ADDITIONAL RESULTS AND DISCUSSION

Table SI1. Composition of the synthesized binary and ternary NSs determined from ICP-OES and EDS.

Feed ratio	Actual composition
Bi_2Se_3	Bi_2Se_3
Bi_2Te_2Se	$Bi_2Te_{2.04}Se_{0.96}$
$Bi_2Te_{2.4}Se_{0.6}$	$Bi_2Te_{2.43}Se_{0.57}$
$Bi_2Te_{2.475}Se_{0.525}$	$Bi_2Te_{2.53}Se_{0.47}$
$Bi_2Te_{2.55}Se_{0.45}$	$Bi_2Te_{2.55}Se_{0.45}$
$Bi_2Te_{2.625}Se_{0.375}$	$Bi_2Te_{2.61}Se_{0.39}$
$Bi_2Te_{2.7}Se_{0.3}$	$Bi_2Te_{2.79}Se_{0.21}$
Bi_2Te_3	Bi_2Te_3

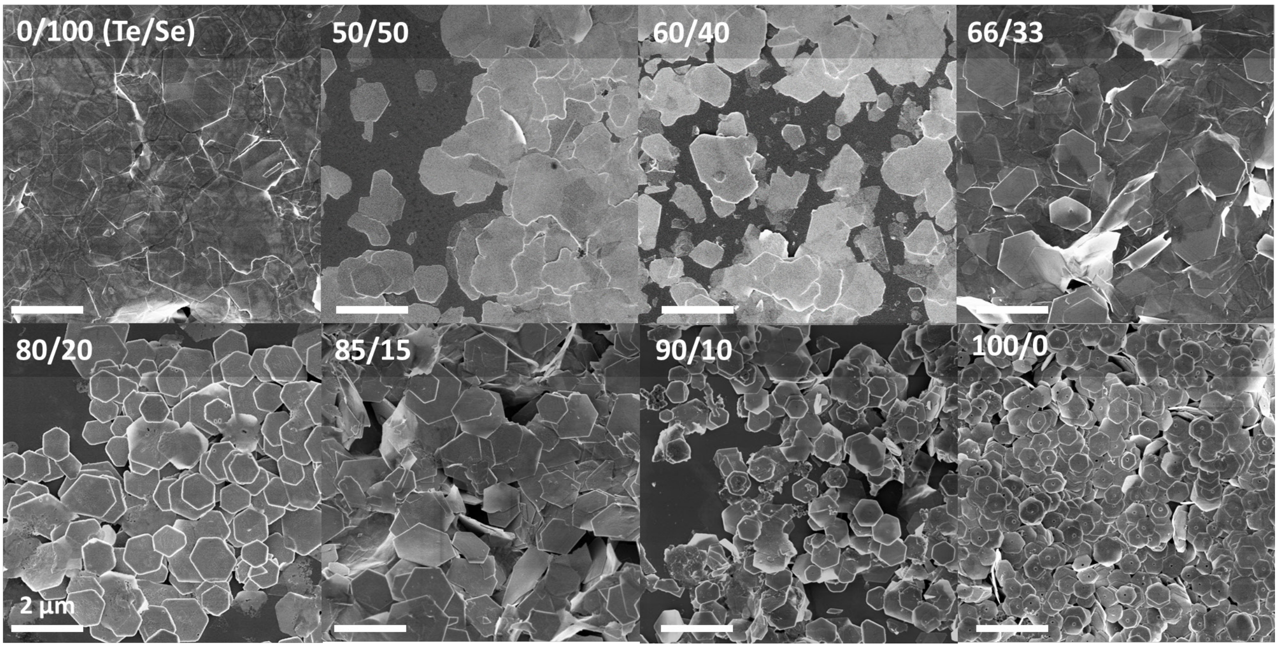

Figure SI 4. SEM images of some binary and ternary NSs. All scale bars are 2 µm.

The formation of $Bi_2Te_{2.55}Se_{0.45}$ NSs was monitored via SEM (Figure SI 5). The first change in the reaction mixture occured at approximately 160°C when the color turned black. At this temperature the yield of the product was very low, but at 170°C the first sample could be taken. Subsequentally after that samples were taken upon reaching 190°C and after 5, 15, 30, 60, 120, and 180 min at 190°C.

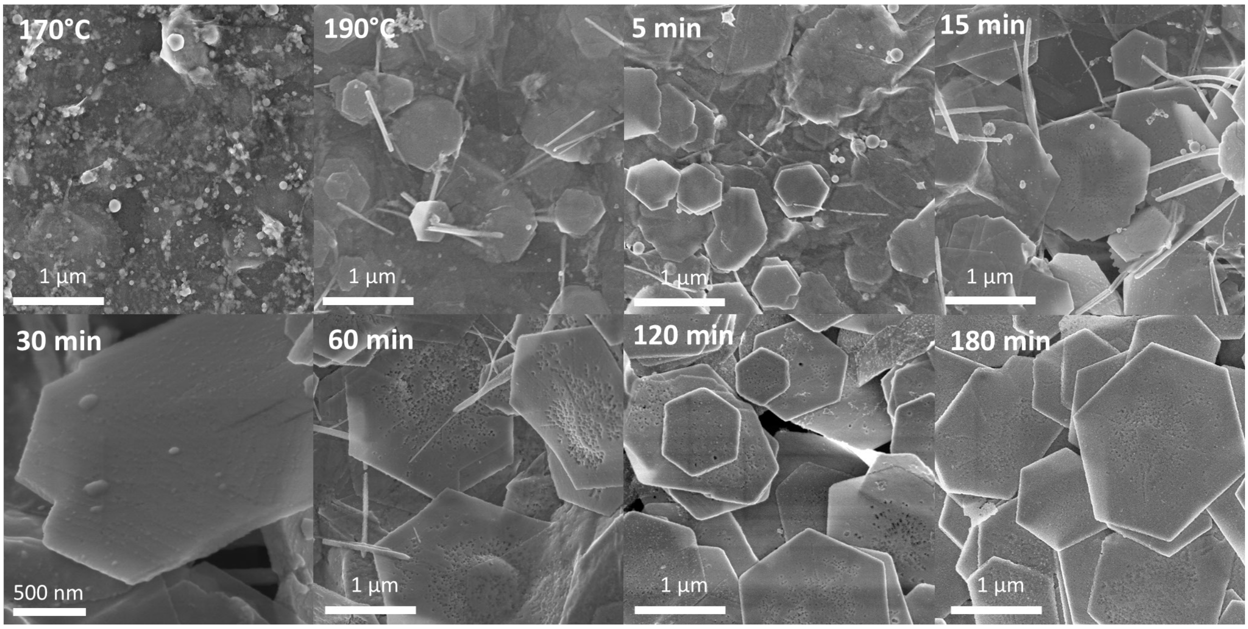

Figure SI 5. SEM images showing the evolution of $Bi_2Te_{2.55}Se_{0.45}$ NSs during the synthesis: samples taken after reaching 170°C, and 190°C, as well as after 5, 15, 60, 120, 180 min of the reaction at 190°C.

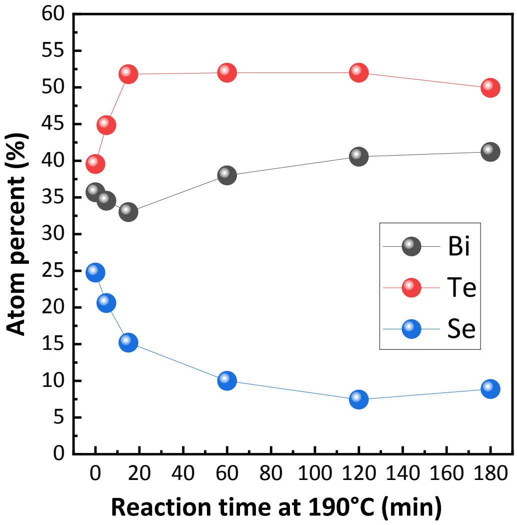

Figure SI 6. EDS results from samples taken during the $Bi_2Te_{2.55}Se_{0.45}$ NS growth.

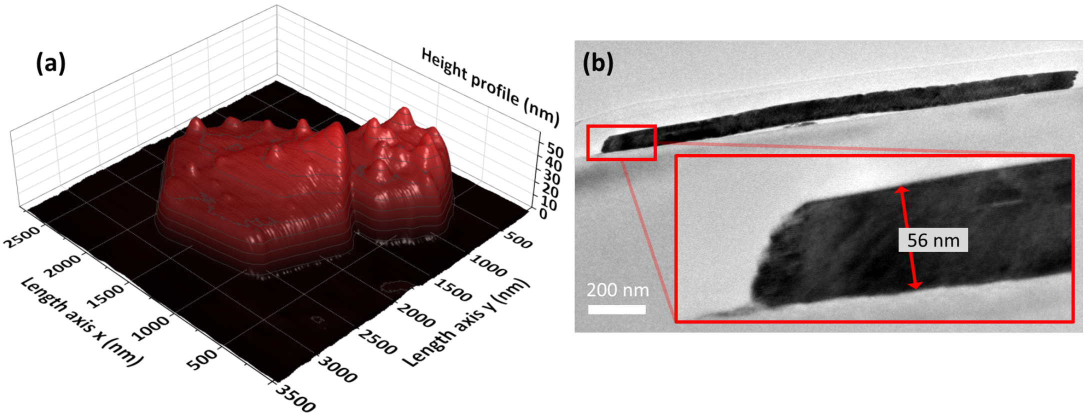

Figure SI 7. AFM (a) and TEM (b) images showing the thickness of the heterostructured $Bi_2Te_{2.55}Se_{0.45}$ NSs: AFM of two adjacently positioned NSs (a), and TEM image of a lamella cut from a $Bi_2Te_{2.55}Se_{0.45}$ NS (b).

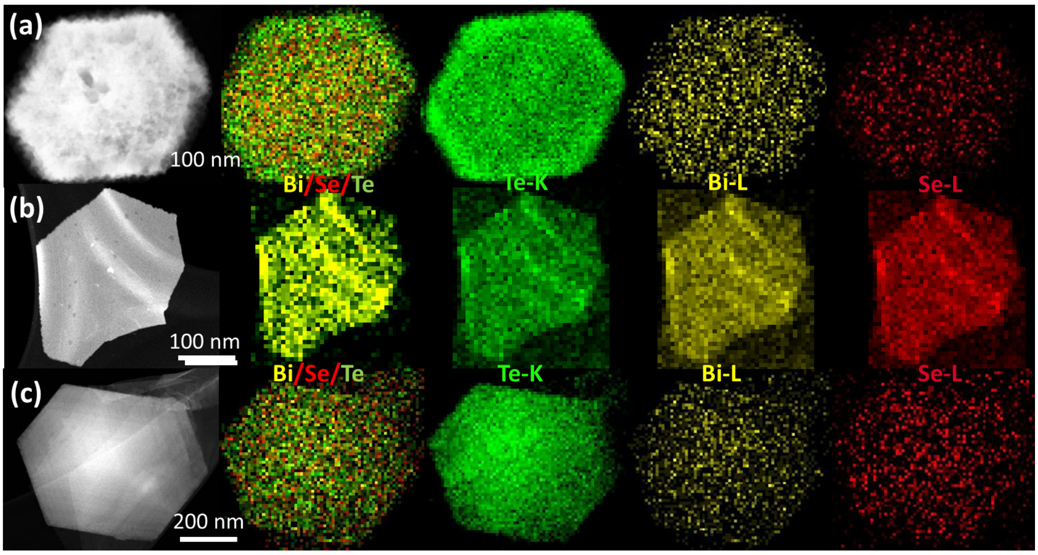

Figure SI 8. HAADF-STEM images and EDS element mapping of $Bi_2Te_{2.4}Se_{0.6}$, $Bi_2Te_{2.7}Se_{0.3}$ and Bi_2Te_2Se NSs. The distribution of Se and Te across the NSs reveal core/shell structure in $Bi_2Te_{2.4}Se_{0.6}$ NSs (a), and a homogeneous distribution for $Bi_2Te_{2.7}Se_{0.3}$ (b) and Bi_2Te_2Se compositions (c).

In XPS characterization accompanied with sputtering experiments we focused primarily on the signals from Bi, Se, Te and O (Figures SI9 and SI10). Signals from C and N were detected in the samples due to PVP coordinating on the surface of the NSs. In the Bi 4*f* spectrum before sputtering of the as-synthesized binary NSs, Bi^{3+} (158 eV) characteristic for the bismuth chalcogenides Bi_2Se_3 and Bi_2Te_3, oxide species (159 eV), as well as metallic bismuth Bi^0 (157 eV) can be distinguished. In all samples the Bi^{3+} component is distinct and accompanied by an oxide band (Figure SI9). The Bi^{3+} signal dominates after sputtering, although the peak broadens which can occur due to the formation of defects (see Figure SI10). After sputtering, the two strong components of the Bi 4*f* signal can be attributed to Bi_2Te_3 or Bi_2Se_3 (157.6 eV) and bismuth oxide (159.0 eV), while the weak component seen as a shoulder at 156.9 eV can be metallic Bi^0, which is either formed by the sputtering process or it is present initially in the as-synthesized NSs being covered by the oxide layer. For the pristine Bi_2Te_3 NSs before sputtering, the signal at 160.5 eV can be deconvoluted and assigned to $Bi(OH)_3$.

Regarding the Se 3*d* signals in pure Bi_2Se_3 sample before sputtering, three deconvoluted components can be assigned to Bi_2Se_3/Se^{2-} (53.4 eV), Se^0 (54.5 eV), and SeO_x (58.5 eV). It was observed that the relative intensity of the oxide component of Te 3*d* is stronger than that of Bi. The Te 4*d* and Te 3*d* signals are sharp in the XPS spectra of the samples. In the Se signal of Bi_2Se_3 NSs the oxide component is much weaker than the corresponding oxide band for Te in Bi_2Te_3. These results suggest, that Bi_2Te_3 NSs are more prone to oxidation than the corresponding Bi_2Se_3.

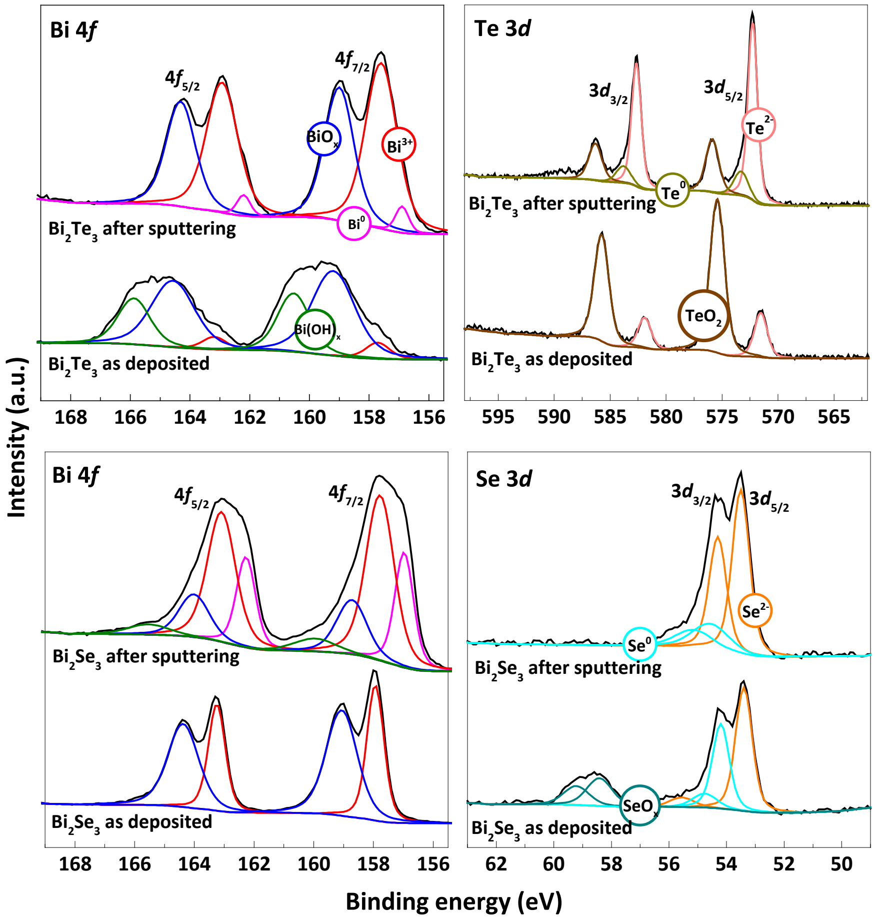

Figure SI 9. XPS data of Bi_2Te_3 and Bi_2Se_3 samples before and after sputtering.

The sputtering significantly reduces oxide signals, as can be seen from Bi, Te and Se spectra in Figure SI 9. In the Bi_2Se_3 sample the Se^{2-} contribution becomes more pronounced, while Se^{4+} disappears. For all the samples, the Te 4*d* bands corresponding to $Bi_2Te_{3-x}Se_x$ preserve their shape and position well after sputtering. The residual signal of oxide species seen in the Te 4*d* spectrum may come from the edge area of the sheets, which are less affected by the sputtering. In analogy to the Te 4*d* spectra, in the Te 3*d* signal three deconvoluted components can be assigned to Bi_2Te_3/Te^{2-} (572.2 eV), Te^0 (573.3 eV), and TeO_2 (576.0 eV).[4] Sputtering of the Bi_2Te_3 sample also reduces the oxide content, but the amount of arising metallic Bi is much smaller than in the Bi_2Se_3 NS sample. The reason for that may be either a difference in thickness of the initial oxide shell or/and a different behavior of Se and Te towards sputtering. During sputtering SeO_2 is removed while Se^{2-} signal remains strong, with a minor Se^0 contribution, and oxygen is removed almost completely. The O 1*s* spectra after sputtering become similar in all samples. The remaining signal is probably from residual oxide at the edges of the NSs. To fit the O 1*s* signal, at least two components are needed which correspond to tellurium oxide and bismuth oxide. The XPS results emphasize for all samples, that the NSs are slightly oxidized on the surface, forming a thin oxide shell.

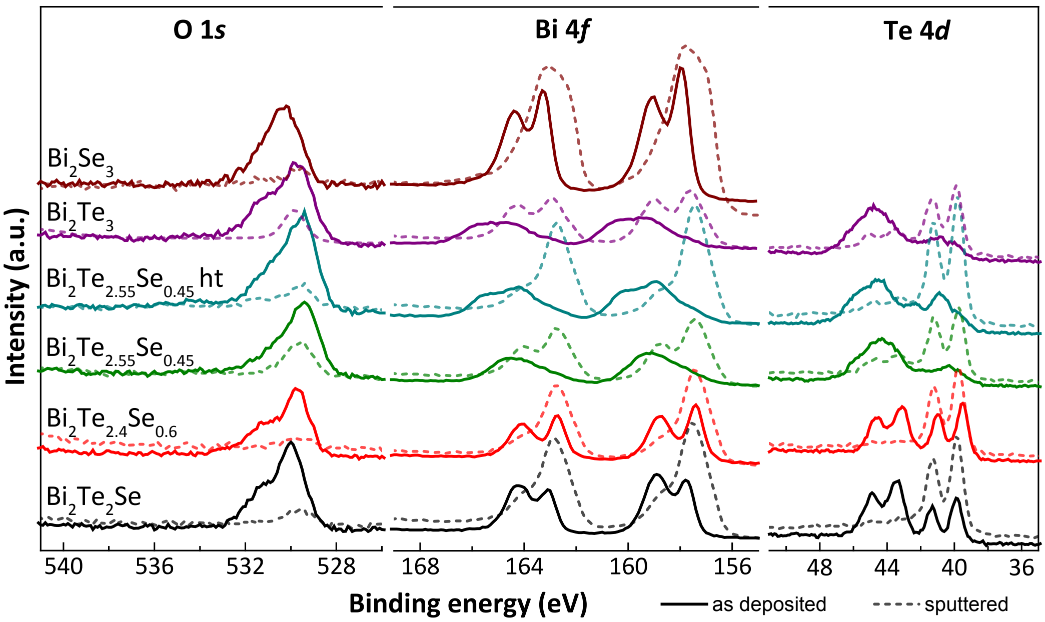

Figure SI 10. XPS data of different NS samples before and after sputtering.

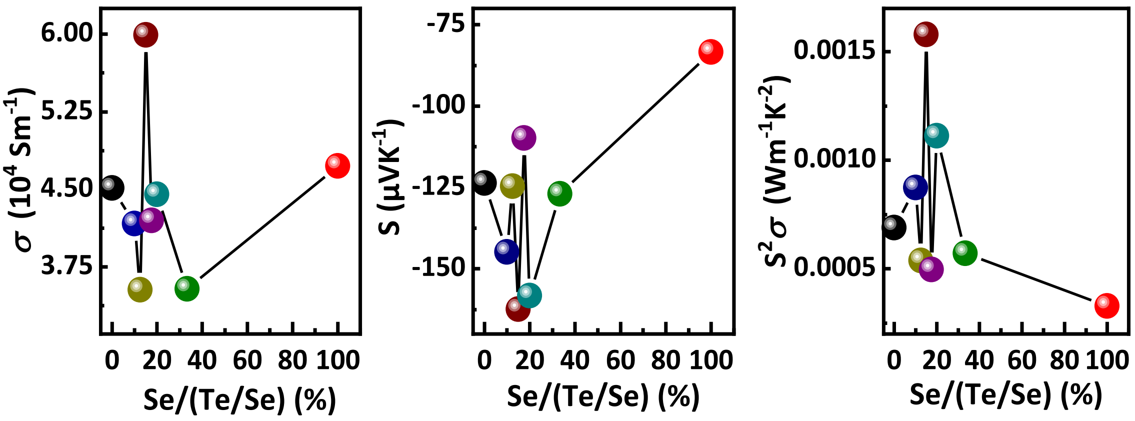

Figure SI 11. Electrical conductivity, Seebeck-coefficient and power factor *vs.* Se content. Samples shown as an overview over different compositions were prepared by method B.

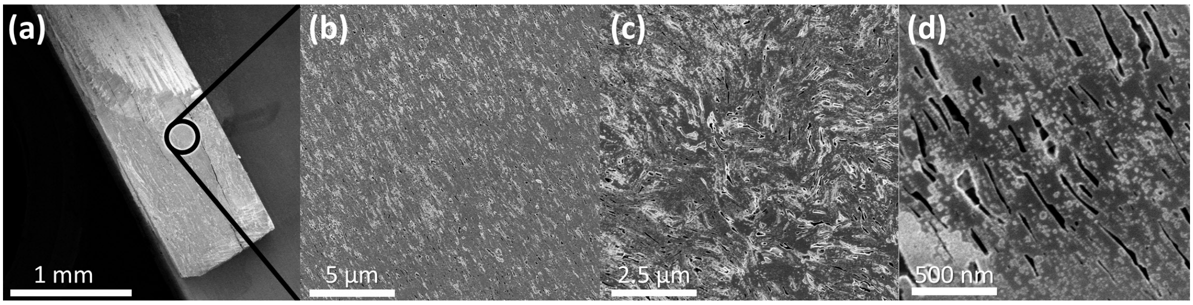

Figure SI 12. SEM images of a sintered Bi_2Te_3 pellet. The pellet (a) is cut for electrical transport measurement and etched by He-plasma to reveal the cross-section structuring at different magnifications (b–d).

A drawback of etching by plasma is the large amount of energy that is exerted locally on the sample, which will fuse the NSs together, giving a false image, where it seems as if most of the nanostructure was lost after sintering. Therefore, mechanically breaking samples and subsequent characterization by SEM can give a different picture of the inner arrangement (Figure SI 13). Here, it can be clearly seen that the grains of the NSs are preserved after preparation, proving the nanostructuring after sintering and showing stacked NSs.

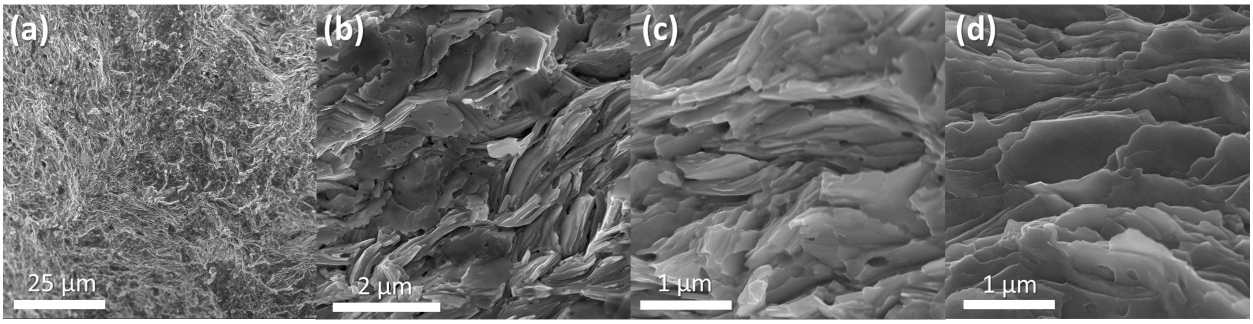

Figure SI 13. SEM images of a sintered $Bi_2Te_{2.55}Se_{0.45}$ pellet which was broken mechanically using a razorblade showing the cross-section, orientation, and microstructure of the NSs at different magnifications.

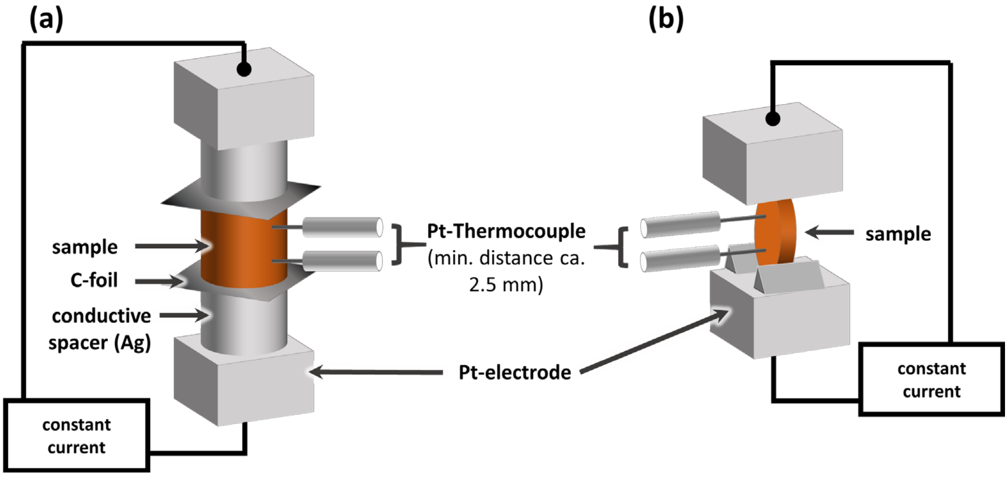

Figure SI 14. Schematic of cross-plane and in-plane sample positioning inside LSR-3. Measurement of cross-plane can be done from sintered samples with a height above 3 mm (a), while extracting in-plane measurement data is done from thin pellets, possible directly from the thin cylindrical geometry (b).

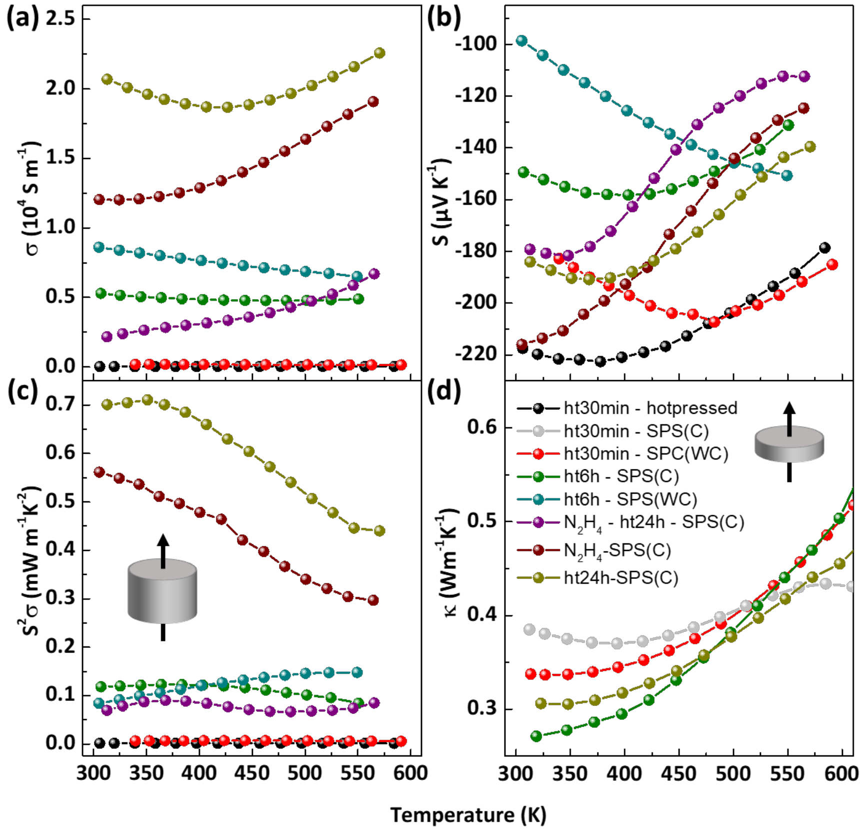

Figure SI 15. Cross-plane thermoelectric characterization of differently sintered $Bi_2Te_{2.55}Se_{0.45}$ pellets: (a) electrical conductivity, (b) Seebeck-coefficient, (c) calculated power factor, and (d) thermal conductivity.

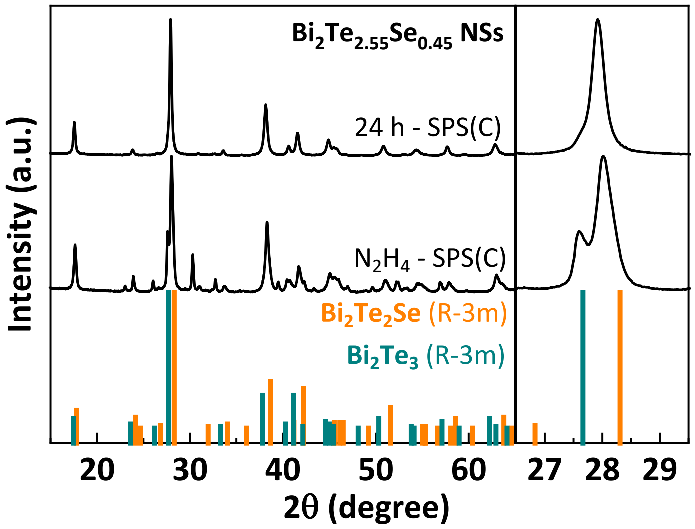

Figure SI 16. XRD of $Bi_2Te_{2.55}Se_{0.45}$ powders produced by milling broken pieces of spark plasma sintered pellets (method A) in an agate mortar. The diffraction patterns in both samples reveal the existence of inhomogeneous crystal phase instead of single-phase reflexes due to complete alloying. The asymmetry of the reflex in the sample that was annealed for 24 h is attributed to the evolved compositional gradient across the NSs.

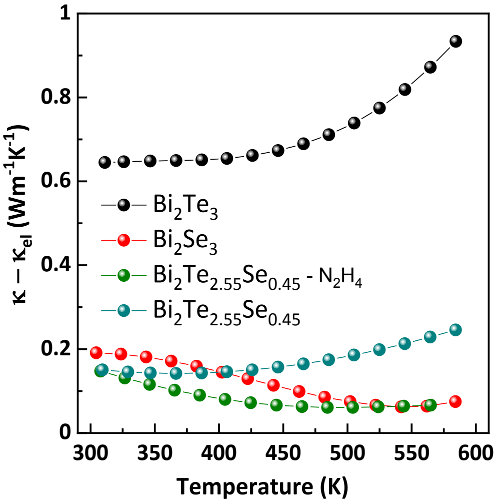

Figure SI 17. Estimated lattice thermal conductivity κ - κ_{el} for samples that were measured perpendicular to the pressing direction (comparison to Figure 8 in the main text). The κ_{el} values were calculated using Wiedemann-Franz-law. The Lorenz-number was estimated with $1.8{\cdot}10^{-8}$ W S^{-1} K^{-2}.

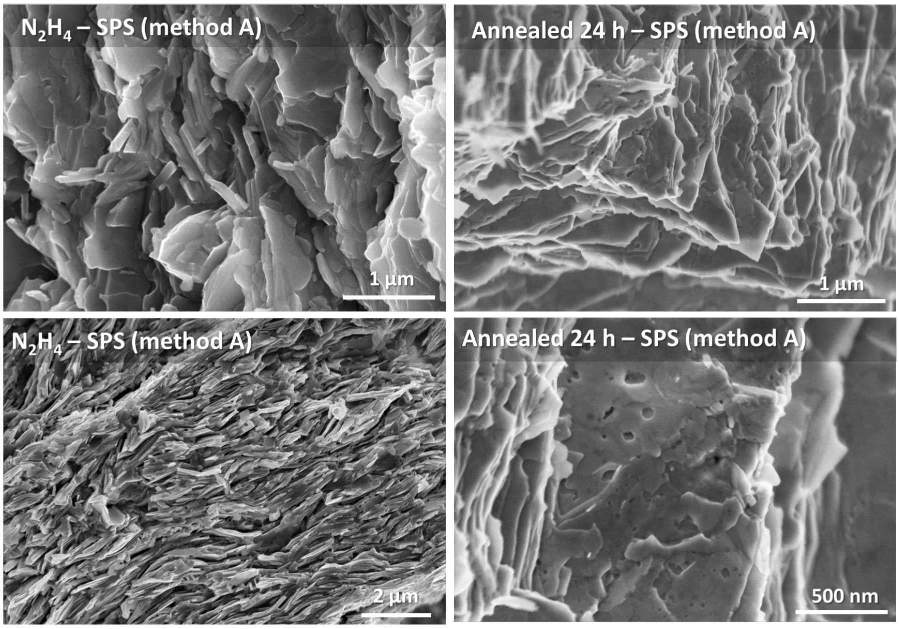

Figure SI 18. SEM images of broken pieces of $Bi_2Te_{2.55}Se_{0.45}$ sample pellets.

REFERENCES

1. M. Hong, T. C. Chasapis, Z.-G. Chen, L. Yang, M. G. Kanatzidis, G. J. Snyder, J. Zou, *Nano Lett.* **2014***, 14*, 6547.
2. Akselrud, L. Grin, Y. *J. Appl. Crystallogr.* **2014**, *47,* 803.
3. M. Hong, T. C. Chasapis, Z.-G. Chen, L. Yang, M. G. Kanatzidis, G. J. Snyder, J. Zou, *ACS Nano* **2016**, *10*, 4719.
4. www.xpsfitting.com/search/label/tellurium (2019/12/03).
